# Lentinan inhibits colorectal cancer stemness by binding CD133 and suppressing the CD133/p85/p-AKT signaling axis

**DOI:** 10.3389/fphar.2025.1725716

**Published:** 2025-12-10

**Authors:** Yan Liu, Lufei Yang, Junxi Liu, Zihao He, Jingyi Wang, Yu Zhang, Kaiping Wang, Jinglin Wang

**Affiliations:** 1 Department of Pharmacy, Union Hospital, Tongji Medical College, Huazhong University of Science and Technology, Wuhan, China; 2 Hubei Province Clinical Research Center for Precision Medicine for Critical Illness, Wuhan, China; 3 Hubei Key Laboratory of Nature Medicinal Chemistry and Resource Evaluation, Tongji Medical College of Pharmacy, Huazhong University of Science and Technology, Wuhan, China; 4 Hubei Key Laboratory of Natural Active Polysaccharides, Union Hospital, Tongji Medical College, Huazhong University of Science and Technology, Wuhan, China; 5 State Key Laboratory of Neurology and Oncology Drug Development, Nanjing, China

**Keywords:** CD133, colorectal cancer, cancer stem cell, shiitake β-glucan, stemness

## Abstract

**Background:**

Colorectal cancer (CRC) ranks third in the global cancer incidence rate worldwide. Cancer stem cells (CSCs) are key drivers of CRC recurrence, metastasis, and therapy resistance, while therapies against them remain limited. Lentinan is widely recognized for its strong immune-enhancing and broad-spectrum directly antitumor activities, however, whether it has potential for cancer stemness remains unknown.

**Methods:**

The CRC cell lines HCT116 and SW620 were selected for pharmacodynamic investigation. Furthermore, tumor sphere formation and limiting dilution assays were used to assess the impact of SLNT on stemness of CRC. Using cell sorting alongside cluster of differentiation 133 (CD133) knockdown and overexpression experiments, the key molecular targets of SLNT in exerting anti-CRC effects were identified. To further elucidate the underlying molecular mechanism, we performed localized surface plasmon resonance (LSPR), cellular thermal shift assay (CETSA), and molecular docking simulations. Additionally, western blot analysis was conducted to validate the key signaling molecules involved.

**Results:**

Our results demonstrated that lentinan significantly suppressed the proliferation and stemness of CRC cell lines HCT116 and SW620. We identified CD133 as a critical functional target and confirmed a direct binding interaction between lentinan and CD133. Finally, we revealed that lentinan suppresses stemness by inhibiting the CD133/ phosphatidylinositol 3-kinase 85 kDa regulatory subunit (p85)/ phosphorylated AKT serine/threonine kinase (p-AKT) signaling axis in CRC.

**Conclusion:**

Our findings not only shed new light on the anti-tumor mechanism of lentinan, highlighting the scientific potential of natural polysaccharides in cancer treatment, but also offers new options for the clinical drug therapy of CRC.

## 1 Introduction

CRC poses a significant global public health challenge, representing the third most frequently diagnosed malignancy and the second leading cause of cancer-related mortality (Eng et al., 2024; Han et al., 2024). The high incidence and mortality rates of CRC remain a major concern in global public health. Statistical data shows that the incidence rate of CRC was 10.7% and the mortality rate was 9.5% in 2020, and it is predicted that the global burden of CRC will continue to increase until 2050 (Morgan et al., 2023; Zhou et al., 2025). Currently, chemotherapy remains an important strategy for CRC treatment (Benson et al., 2021; Benson et al., 2024). Although chemotherapy can impede CRC progression to some extent, most treated patients fail to achieve complete tumor eradication, with prolonged administration of chemotherapeutic agents such as 5-fluorouracil and oxaliplatin not only accompanying side effects but also resulting in adverse outcomes such as drug resistance, recurrence, and metastasis (Souglakos et al., 2006; Del Rio et al., 2007; He et al., 2025). The poor therapeutic prognosis is attributed to the inability of conventional chemotherapy to eliminate CSCs, which drive tumor persistence and metastatic dissemination (Wang et al., 2020; Xie et al., 2022). CSCs comprise a subpopulation of tumors possess the abilities of immortality, dormancy, self-renewal, and multidirectional differentiation capacities, responsible for driving tumor heterogeneity, growth, metastasis, recurrence, and therapeutic resistance (Nassar and Blanpain, 2016). Mounting evidence suggests that prolonged administration of first-line chemotherapeutic agents not only fails to suppress CSCs but paradoxically induces their activation and enrichment among residual tumor cells, thereby amplifying tumor cell stemness (Giuliano et al., 2011; Cho et al., 2020; Jo et al., 2022). It is therefore critical to discover a drug with good therapeutic effect, few side effects, and inhibitory effect on CSCs for the clinical treatment of CRC.

Natural polysaccharides with anti-tumor activity have attracted widespread attention owing to their high efficiency, minimal cytotoxicity and multi-target regulatory properties (Khan et al., 2019). Accumulating evidences further indicates that polysaccharides hold promising potential for the treatment of CSCs. For instance, Huaier polysaccharide, Astragalus polysaccharide, and fucoidan have been reported to suppress the stemness properties of breast cancer, melanoma, and bladder cancer CSCs, respectively, thereby exerting antitumor effects (Hu et al., 2019; Gong et al., 2022; Sung et al., 2022). Lentinan, a kind of β-glucan, is the primary bioactive component of *Lentinus edodes* and is well recognized for its potent immune-enhancing and antitumor activities (Rao et al., 2021). Beyond its antitumor effects mediated by immune modulation, a growing body of studies in recent years has demonstrated that lentinan exerts broad-spectrum, immune-independent direct antitumor effects against various cancer cell types, and preliminary investigations into its underlying direct antitumor mechanisms have been conducted. In current studies investigating the direct antitumor mechanisms of lentinan, most research has demonstrated that it exerts tumor-suppressive effects primarily via triggering apoptosis of cancer cells, blocking the cell cycle progress, and suppressing angiogenesis (Deng et al., 2018; Hu et al., 2023). These reports mainly focused on the direct cytotoxic mechanisms of lentinan on differentiated cancer cells, whereas its inhibitory potential against CSCs remains unclear. Our previous work demonstrated that lentinan suppresses the stemness of breast cancer cells (Gu et al., 2022), suggesting that it may exert a similar effect on colorectal cancer cells.

CD133 is a pentameric transmembrane cholesterol-binding glycoprotein that localizes to the plasma membrane, which is widely expressed in solid tumors and hematological malignancies (Gisina et al., 2023). Furthermore, CD133 serves as a biomarker for CSCs, including those of CRC, and is involved in regulating multiple key signaling pathways associated with tumor fate. Beyond its utility as a marker, CD133 possesses intrinsic molecular functions. There is growing consensus that CD133 is critically involved in the self-renewal and tumorigenic capacity of CSCs (Mak et al., 2012; Liang et al., 2021; Moreno-Londoño and Robles-Flores, 2024). Consequently, targeting CD133 has become a promising anticancer therapeutic strategy. Firstly, it can directly bind to CD133 protein and deliver genes or drugs through various vectors, thereby reducing the number of CD133^+^ CSCs, and exerting anti-tumor effects (Dianat-Moghadam et al., 2023). Additionally, by inhibiting the expression of CD133, it disturbs key downstream signaling pathways, such as wingless/integrated (Wnt)/beta-catenin signaling pathway, epidermal growth factor receptor (EGFR)/AKT serine/threonine kinase (Akt)/mammalian target of rapamycin (mTOR) signaling cascade, and CD133/programmed cell death ligand 1(PD-L1) signaling axis, thereby impairing stemness properties and inhibiting tumor progression (Mak et al., 2012; Liang et al., 2021; Xu et al., 2023; Wang et al., 2025). Although current studies have found that some polysaccharides suppress the stemness of tumor cells, the research on the molecular mechanism by which these polysaccharides restrain the characteristics of tumor stem cells is relatively limited.

Our previous study revealed that lentinan notably restrained the proliferation of CRC cell line HT-29 and CD133 protein expression, suggesting that lentinan has the potential for suppressing the stemness of CRC. Nevertheless, the specific role of CD133 in this process and the underlying molecular mechanisms require further investigation. Natural polysaccharides, owing to their high molecular weight and numerous side chains, have the conditions to bind to various receptors or proteins. For instance, hydroxyl-rich β-glucan can bind with pattern recognition receptors (PRRs) such as Dectin-1, toll-like receptor 2 (TLR2), toll-like receptor 4 (TLR4), toll-like receptor 6 (TLR6), and complement receptor 3 (CR3), to activate the immune response (Chan et al., 2009). Hyaluronic acid (HA) can bind to the cell membrane glycoprotein cluster of differentiation 44 (CD44), which is considered a cancer stemness marker, thereby transducing intracellular signaling and regulating a variety of biological activities of cancer cells (Dulal et al., 2018; Weng et al., 2022; Wold et al., 2024). β-glucan Hericium polysaccharide was reported to hinder CD133 expression and suppress lung cancer growth when used in combination with aspirin (Lu et al., 2019). Here, we hypothesized that lentinan might also bind to the CD133 on the surface of CRC cells and inhibit its expression, thus affecting the downstream signal pathway and inhibiting the stemness of CRC cells, thereby exerting anti-tumor effects. First, we evaluated lentinan’s antitumor effects across experimental models. Subsequently, we assessed the influence of lentinan on the stemness of CRC cells using tumor sphere formation assays and limiting dilution assays. Then, we validated the function of CD133 and its role in the anti-colorectal cancer effect of lentinan through gene knockdown and overexpression experiments. Additionally, we employed experiments including LSPR, CETSA, molecular docking simulation assay, flow cytometry sorting assays and Western blot assay to elucidate in-depth the mechanism by which lentinan suppresses stemness and to identify the involved downstream signaling pathways. In brief, our research aims to explore the role of lentinan against the stemness in CD133^+^ CRC cells and to identify the underlying downstream pathways.

## 2 Materials and methods

### 2.1 Materials

HCT116, SW620 and SW480 cells were procured from Procell (Hubei, China). B27 (17504044) was purchased from Invitrogen, human epidermal growth factor (hEGE, AF-100-15-100) and human fibroblast growth factor (hFGF, 100-18B-50) were purchased from PeproTech (London, United Kingdom). DMEM, McCoy’s 5a medium, and fetal bovine serum (FBS), penicillin-streptomycin were purchased from Gibco Life Technologies Co. (Grand Island, New York, United States). We purchased antibodies against CD133 (ab278053), p85 (ab191606), p-Src (ab133460), p-AKT (4060T) for Western blot from Abcam (Cambridge, MA, United States) and Cell Signaling Technology (CST, Danvers, MA, United States). CD133/2-PE (130-112-748) were purchased from MiltenyiBiotec (Cologne, Germany). Crystal violet was purchased from Servicebio (Wuhan, Hubei province, China). Human recombinant protein CD133 was purchased from ACROBiosystems (Cat. No.CD3-H82Q8, United States). Ultra-Low Attachment culture plates were purchased from Corning (New York, United States). Puromycin dihydrochloride was purchased from Beyotime Biotechnology (Shanghai, China).

### 2.2 Preparation and structural characterization of *Lentinus edodes* polysaccharides

Lentinan was extracted, isolated, and purified from the dried fruit bodies of *Lentinus edodes* originating from Fangxian (Hubei Province, China) using an established protocol (Wang et al., 2013). The lentinan obtained by the hot water extraction method named SLNT, was applied to this study. The structure of SLNT is consistent with our previous study, indicating that the polysaccharide is stable in properties (Zhang et al., 2023). Detailed information about polysaccharide extraction can be found in the Supplementary File 1.

### 2.3 Tumor sphere formation and limiting dilution assay

In this experiment, we used serum-free medium DMEM/F12 containing 2% B27, 20 ng/mL hEGF, and 20 ng/mL basic hFGF to culture cells. 1,000 cells were seeded in a 6-well low attachment culture plate (Corning, United States) and cultured with 0, 200, 400, 800 μg/mL SLNT solution for 7 days. After tumor spheres formed, spheres were photographed under microscope. For limiting dilution assay, 1, 10, 50, 100, 250, 500 cells/well or 1, 10, 50, 100, 500, 1,000 cells/well were seeded in a 96-well low attachment culture plate for 7 days. Spheres number was counted under microscope and the sphere synthesis efficiency were analyzed via Extreme Limiting Dilution Analysis (Hu and Smyth, 2009) (ELDA, http://bioinf.wehi.edu.au/software/elda).

### 2.4 Animal models

We purchased 5-week-old BALB/c male nude mice from HFK Bioscience (SCXK 2019-0008, Beijing, China). The experiment began after all mice were adaptively fed for 7 days. HCT116 cells, shNC/shCD133 HCT116 cells, and OENC/OECD133 SW480 cells (6–9 × 10^6^) were injected subcutaneously into the right side of the mice. We randomly divided the mice into the following groups: NC (0.9% NaCl solution) group, 1 mg/kg SLNT group, 5 mg/kg SLNT group, shNC/OENC HCT116/SW480 group, shNC/OENC+1 mg/mL SLNT group, shCD133/OECD133 HCT116/SW480 group, shCD133/OECD133 + 1 mg/kg SLNT group. SLNT or 0.9% NaCl solution was injected via tail vein every 2 days for 10 times. Tumor volume and body weight were recorded at the same time of administration (Tumor volume = 0.5 × length × width^2^). Upon completion of the treatment, the nude mice were executed and the serum, tumor tissue, liver, spleen, and kidneys were removed and preserved for subsequent experiments. Animal studies were conducted under specific pathogen-free (SPF) conditions with strict regulation of temperature (25 °C ± 1 °C) and humidity (50%–60%), on a 12 h light/dark cycle. The mice had free access to food and water. The Institutional Animal Care and Use Committee of Tongji Medical College, Huazhong University of Science and Technology approved all animal experiments (IACUC Number: 3619), which were followed the National Research Council’s Guide for the Care and Use of Laboratory Animals.

### 2.5 Construction of stable transgenic shCD133 HCT116 cells and OECD133 SW480 cells

Lentiviral vector shCD133 expressing shRNAs targeting human CD133 (CD133 sense 5′-GUG​UAC​AGU​AAA​CGG​UGU​AUA​TT-3′), and the control vector shNC. A CD133 overexpression vector was purchased from Genechem (Shanghai, China). CD133 overexpression vector (OECD133) and shCD133 or the negative control vector were incubated with SW480 cells and HCT116 cells for 12 h. Then, the supernatant containing lentivirus was removed and fresh culture medium was added. After 72 h of lentivirus addition, cells were replaced with a puromycin-containing medium instead of the normal medium and continued to be cultured for 2 weeks to screen for stable knockdown or overexpression.

### 2.6 Localized surface plasmon resonance (LSPR) assay

The kinetics of binding between SLNT and CD133 were detected by OpenSPRTM. SLNT and human recombinant protein CD133 were dissolved in PBS. Briefly, the protein CD133 was immobilized on a COOH chip. For the measurement of interaction between SLNT and CD133, different concentrations of SLNT were loaded onto the chip at 20 μg/min. SLNT was bound to the CD133 protein for 240 s and dissociated naturally for 360 s. We processed and analyzed the data using TraceDrawer software (Ridgeview Instruments ab, Sweden) and the One to One analysis model.

### 2.7 Cellular thermal shift assay (CETSA)

The principle and detailed protocol of the CETSA has been reported in previous study (Jafari et al., 2014). In short, HCT116 cells were cultured in 10 cm dishes and grown to 80%–90% confluency. Then, we treated the cells with 1,600 μg/mL SLNT or an equal volume of culture medium for 6 h. Next, we collected the cells and resuspended them in 600 μL PBS containing 1% PMSF. Subsequently, the cells were divided into 6 tubes (100 μL/tube) and heated for 5 min using a series of temperature gradients. After the cells were lysed and centrifuged for 30 min at 4 °C, collected the supernatant for the Western blot assay.

### 2.8 Molecular docking simulation

A molecular docking simulation assay was performed to predict the binding site between SLNT and CD133. Protein structure information was obtained from the RCSB PDB database. AlphaFold crystals were used to simulate human protein CD133 in the database for subsequent analysis (AlphaFold ID: AF-O43490-F1). The structure of SLNT was simulated using Gromacs software and analysed by molecular dynamics simulation in a water molecule environment for at least 90 ns, and finally, the optimal structure was selected for subsequent docking analysis. ZDOCK software was used for docking analysis. The site phase with the most clusters and the lowest energy of each system was selected as the optimal docking structure and the binding site between SLNT and CD133 was speculated.

### 2.9 Statistical analysis

Data analysis was conducted using GraphPad Prism 9, with results presented as the mean ± SD from a minimum of three independent replicates. To determine statistical significance, we employed unpaired two-tailed Student’s t-test for comparisons between two groups. For comparisons involving three or more groups, one-way analysis of variance (ANOVA) followed by Tukey’s *post hoc* test was applied for multiple comparisons, and Dunnett’s *post hoc* test when comparing each group with a certain group. Statistical significance was defined at p < 0.05.

## 3 Results

### 3.1 Characterization of SLNT

**FIGURE 1:**
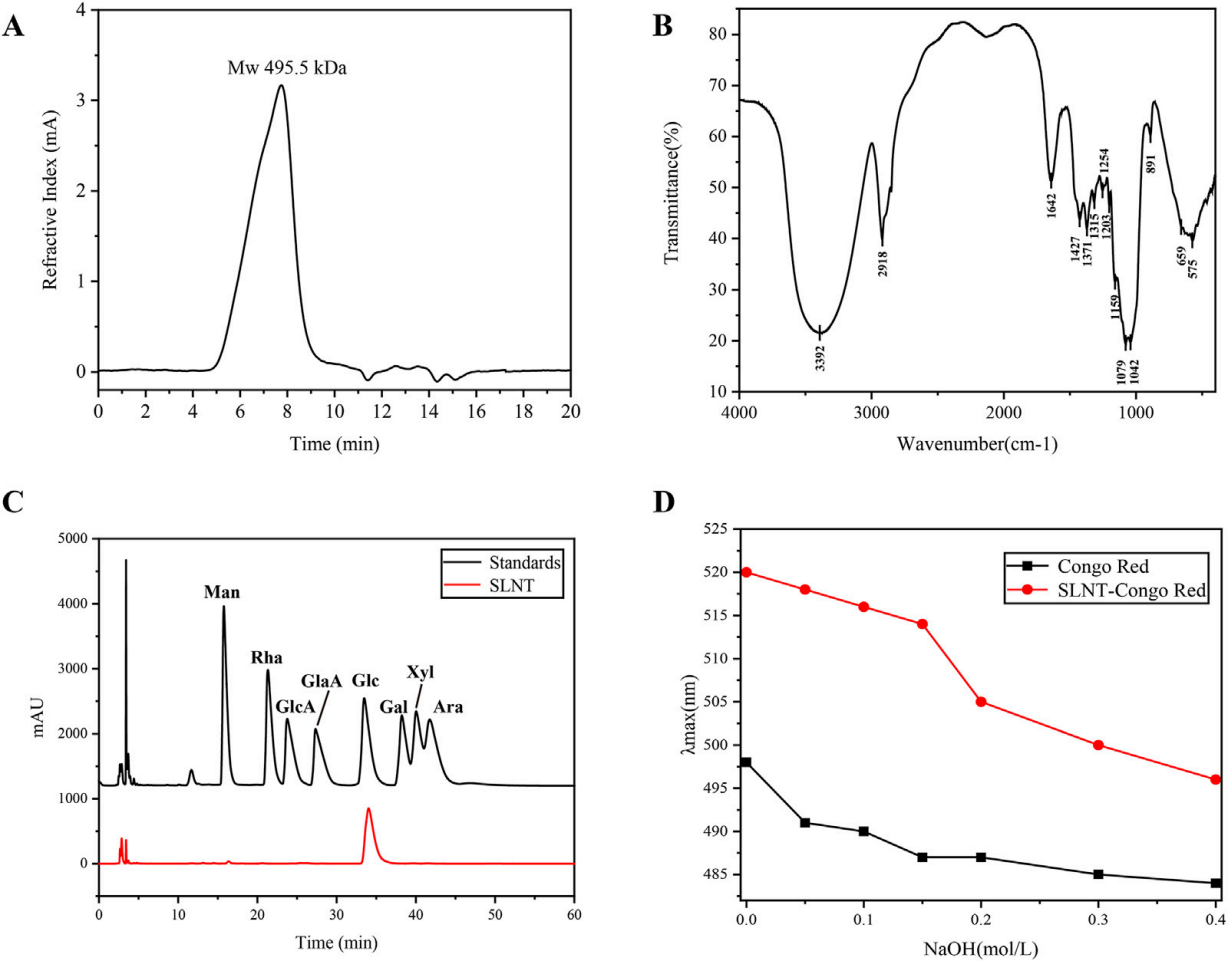
Characterization of SLNT. High-performance liquid chromatography (HPGPC) chromatogram of SLNT **(A)**, Fourier transform infrared spectrometer (FT-IR) spectrum **(B)**, monosaccharide composition **(C)** and Congo red analysis **(D)** of SLNT. Man, D-mannose; Rha, L-rhamnose; GlcA, D-glucuronic acid; GalA, D-galacturonic acid; Glc, D-glucose; Gal, D-galactose; Xyl, D-xylose; Ara, L-arabinose.

The molecular weight of SLNT extracted from *L. edodes* was determined to be 495.5 kDa (Figure 1A). According to Figure 1B, the peak at 3,392 cm^-1^ was assigned to O-H stretching, the peak at 2,918 cm^-1^ was assigned to C-H stretching, and the absorption band at 1,642 cm^-1^ was corresponds to bound water. Three characteristic absorption peaks, at 1,159 cm^-1^, 1,079 cm^-1^ and 1,042 cm^-1^, appeared between 1,160 and 1,010 cm-1, indicating SLNT is a kind of pyranose. The characteristic absorption peak at 891 cm^-1^ indicates that SLNT has a β configuration. The results of Figure 1C revealed that SLNT was composed of D-glucose. The Congo red results showed that SLNT was a type of β-glucan with a triple helix structure (Figure 1D). The results of the methylation analysis of SLNT are shown in Supplementary Figure S1. The types of sugar residues in SLNT are T Glc*p*, 1,3 linked Glc*p*, 1,6 linked Glc*p* and 1,3,6 linked Glc*p*, with a ratio of 2.3:4.3:1.0:2.9. Results confirmed that 1,3-linked Glc*p* constitutes the main backbone structure of SLNT, while the presence of the branch point residue 1,3,6-linked Glc*p*, suggests that SLNT has branched chains. The structural information for SLNT was concordant with our previous findings, which validated our extraction method and the consistency of the identified structure (Wang et al., 2013; Zhang et al., 2023).

### 3.2 The oncogenic effect of SLNT in CRC cell lines both *in vitro* and *in vivo*

**FIGURE 2:**
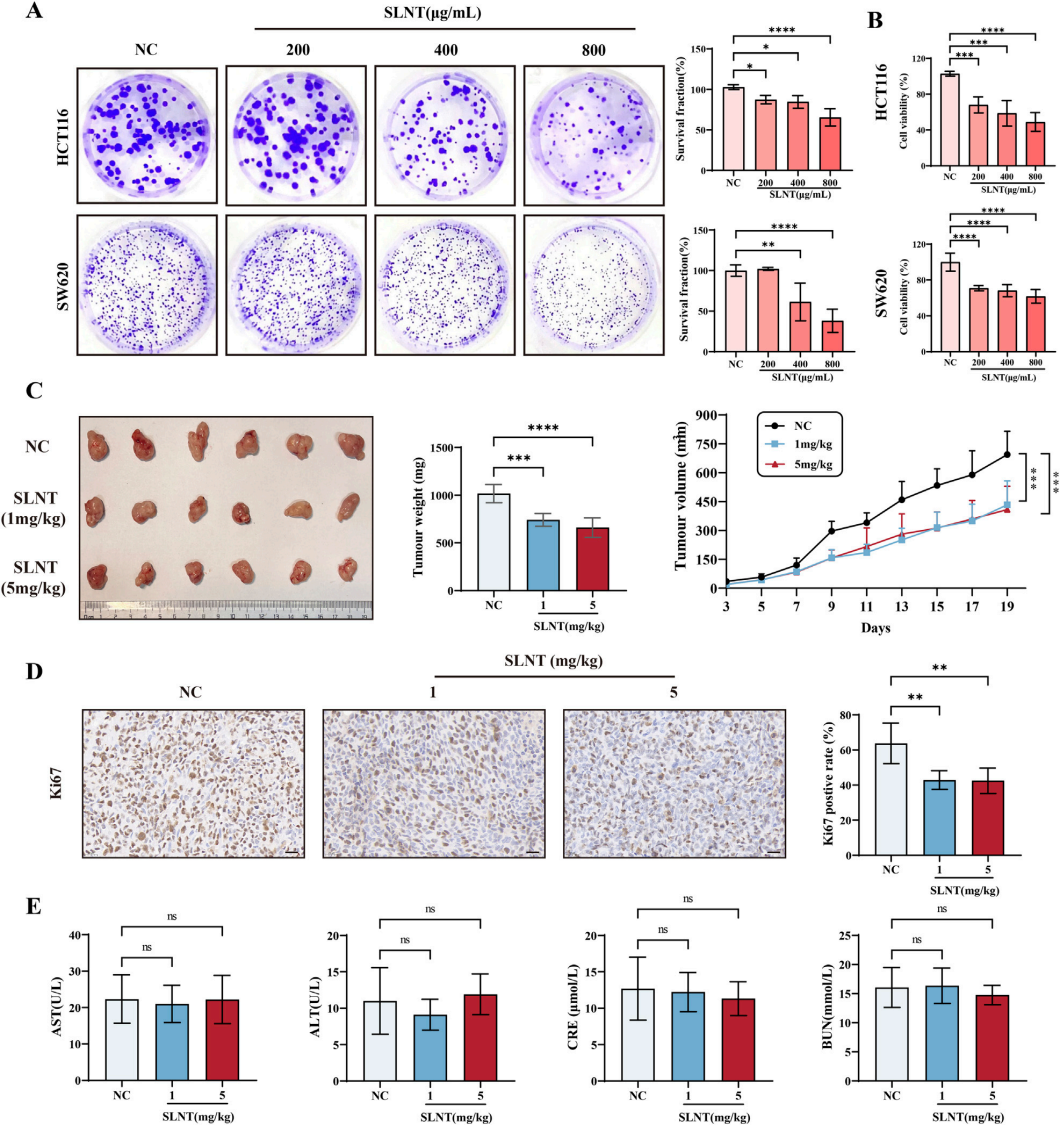
SLNT inhibits CRC proliferation *in vitro*. CRC cell lines HCT116 and SW620 were treated 800 μg/mL different concentration of SLNT for 48 h. Self-renewal ability was detected by clone formation assay **(A)**, and cell viability was detected by CCK-8 **(B)**. Representative pictures of HCT116 tumors, tumor weight, and tumor volume **(C)** of HCT116 xenograft model (n = 6). Immunohistochemistry for Ki67 and semiquantitative analysis of immunohistochemistry in HCT116 tumor-bearing nude mice **(D)**. Serum aspartate aminotransferase (AST), alanine aminotransferase (ALT), creatinine (CRE), blood urea nitrogen (BUN) expression levels in each group of HCT116 tumor-bearing mice **(E)**. n ≥ 3, ^*^ *p* < 0.05, ^**^ *p* < 0.01, ^****^ *p* < 0.0001, ns: no significance.

The cytotoxicity of SLNT to CRC cell lines HCT116 and SW620 was evaluated *in vitro*. As clearly shown in Figures 2A,B, SLNT inhibited the viability of the CRC cell lines in a dose-dependent manner and simultaneously impeded the clonogenicity of the tumor cells *in vitro*. Similarly, we employed an HCT116 xenograft model in nude mice to assess the *in vivo* anti-tumor activity of SLNT. After 1 mg/kg and 5 mg/kg SLNT treatment, tumors in the HCT116 xenograft model exhibited a notable reduction in both weight and volume than those in the control group (Figure 2C), showing a good inhibitory effect of SLNT on CRC cells *in vivo*. Additionly, SLNT treatment significantly hindered tumor cell proliferation, as indicated by a remarkable downregulation of the ki67 marker in tumor tissues (Figure 2D). Meanwhile, there were no significant differences in liver and kidney injury indicators or body weight among the various groups, suggesting that SLNT exhibits no notable toxic or adverse effects on mice (Figure 2E; Supplementary Figure S2). Moreover, we employed the radionuclide technetium-labeled SLNT (99mTc-LNT) protocol, which was successfully established by our research group in previous studies, along with qualitative detection methods to investigate the distribution of SLNT in the tumor tissues of HCT116 xenograft model (Zhang et al., 2018; Dou et al., 2025). It was found that polysaccharide signals could be detected in the tumor site at 15 min, 30 min, and 1 h after intravenous administration (Supplementary Figure S3). This indicates that SLNT may act directly on the tumor site *in vivo* and also provides pharmacokinetic evidence for the direct anti-tumor effect of SLNT *in vivo*.

### 3.3 SLNT suppresses the stemness of CRC cells

**FIGURE 3:**
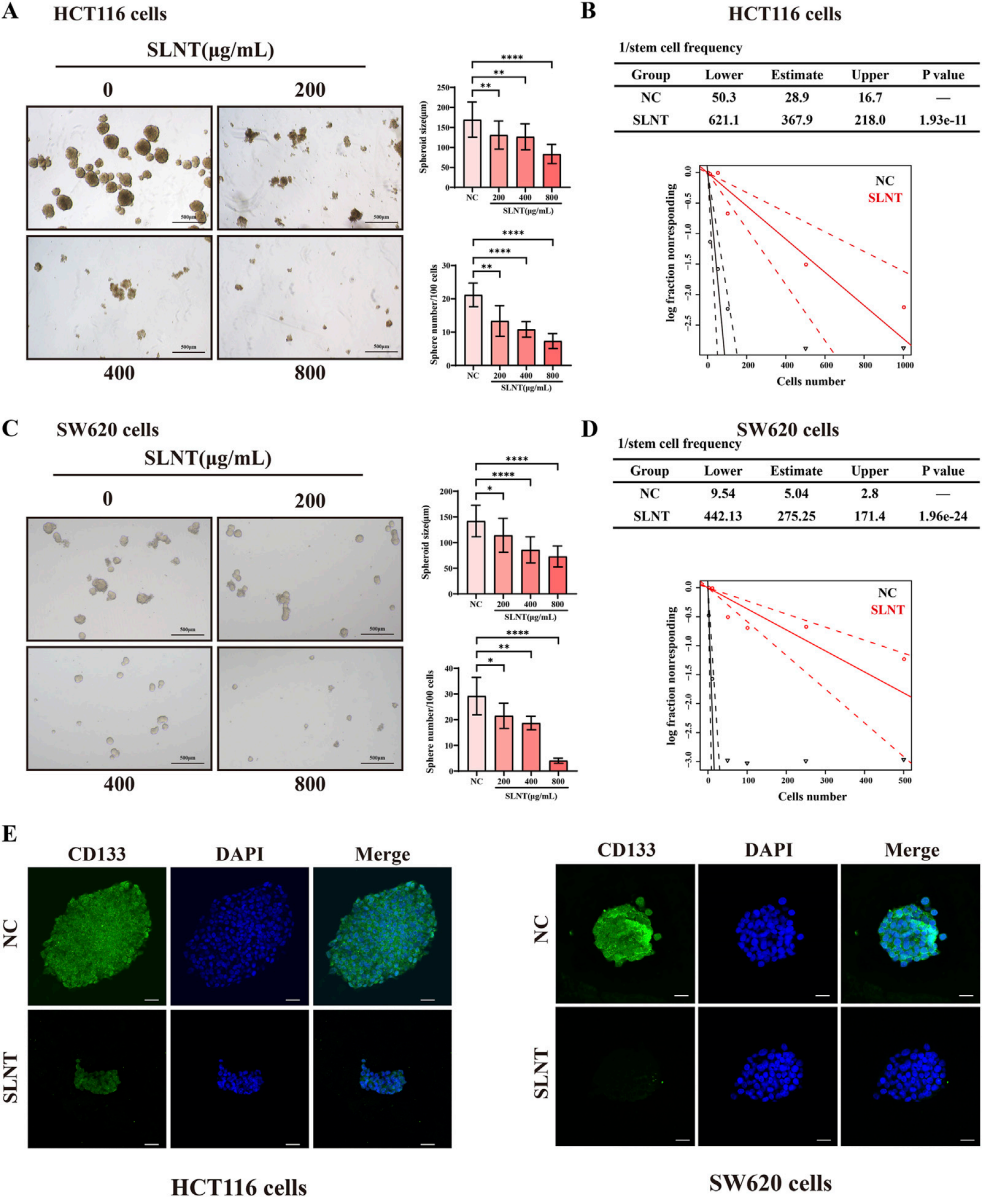
SLNT suppresses the stemness of CRC. Tumor sphere formation **(A,C)** and limiting dilution analysis **(B,D)** of HCT116 and SW620 cells. CD133 expression of HCT116 and SW620 spheres were detected by immunofluorescence assay with or without 800 μg/mL SLNT treatment **(E)**. n ≥ 3, ^*^ *p* < 0.05, ^**^ *p* < 0.01, ^****^ *p* < 0.0001, ns, no significance.

CSCs are the source of tumor proliferation, metastasis, and recurrence. Our group reported that lentinan could inhibit the stemness of breast cancer cells thus hindering tumor recurrence (Gu et al., 2022). To further determine the effect of SLNT on colorectal cancer stemness, a sphere-forming assay and ELDA were performed based on the principle that normal cells cannot survive in stem cell culture medium (Fang et al., 2010; Lathia et al., 2015). In Figures 3A–D, the reduced number and size of spheres indicated that SLNT could destroy the morphology of the cells, impairing the sphere formation abilities of CRC cell lines HCT116 and SW620 cells. Moreover, the expression of CRC stemness marker CD133 in the HCT116 and SW620 spheres was markedly weakened by SLNT, according to the immunofluorescence results (Figure 3E).

### 3.4 CD133 is a key molecule for SLNT to exert its anti-CRC effect

CD133 is widely recognized not only as a canonical marker of stemness but also as a pivotal pro-oncogenic factor. Accumulating evidence has demonstrated that upregulating CD133 expression sustains the core properties of CSCs through the modulation of multiple signaling pathways. Specifically, it regulates cellular processes closely associated with tumorigenesis, including apoptosis and autophagy, thereby supporting cancer cell growth and sustaining disease progression (Simbulan-Rosenthal et al., 2024; Sipos and Műzes, 2024; Hua and Cai, 2025). Thus, it is necessary to evaluate the molecular function of CD133 in CRC and the role it plays in the anti-colorectal cancer effect of SLNT. According to the gene expression profiling interactive analysis (GEPIA) database, the CD133 marker in CRC is prominently upregulated compared to para-carcinoma tissue, and is negatively correlated with disease-free survival and overall survival of CRC patients (Supplementary Figure S4), indicating that CD133 is a reliable carcinogenic factor in CRC.

**FIGURE 4:**
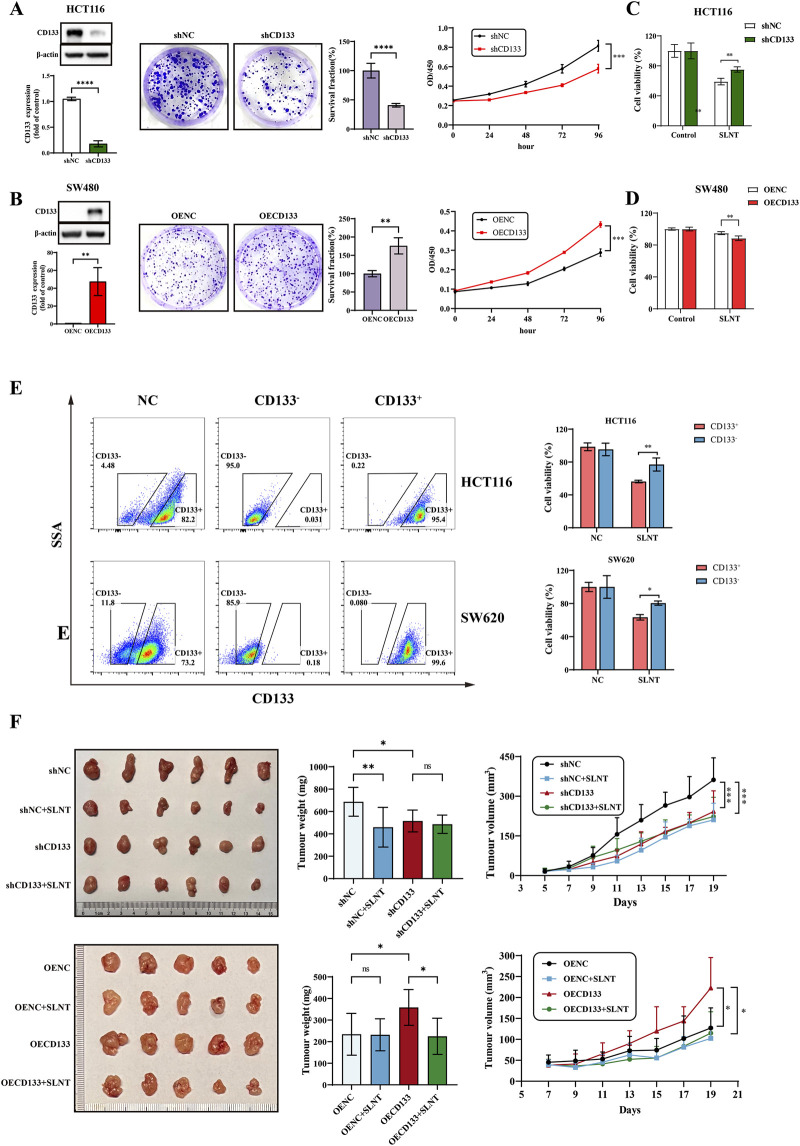
CD133 is a key molecule for SLNT to exert its anti-CRC effect. Cell viability and clone formation capacity were functionally affected by CD133 knockdown **(A)** and overexpression **(B)**. After 800 μg/mL SLNT treated, cell viability of CD133 knockdown HCT116 cells **(C)** and overexpression SW480 cells were detected by CCK-8 **(D)**. Cell viability of CD133^-^ and CD133^+^ fraction in CRC cell lines HCT116 and SW620 were detected with or without 800 μg/mL **(E)**. **(F)** Representative pictures of tumors, tumor weight and tumor volume in each group of shCD133 HCT116 (above) and OECD133 SW480 tumor-bearing mice (below) (n = 8). n ≥ 3, ^*^ *p* < 0.05, ^**^ *p* < 0.01, ^***^ *p* < 0.001, ^****^ *p* < 0.0001, ns, no significance.

Next, we explored the role of CD133 in the anti-colorectal cancer efficacy of SLNT. Here, we selected HCT116 cells with high CD133 expression and SW480 cells with low CD133 expression (Supplementary Figure S5) to conduct gain and loss-of-function studies of CD133 *in vitro* and *in vivo*. Here, we successfully constructed stably transduced CD133-knockdown HCT116 cells (shCD133 HCT116 cells) and CD133-overexpressing SW480 cells (OECD133 SW480 cells), and reported that CD133 promoted the growth and proliferation of CRC cell lines both *in vitro* (Figures 4A,B). Else, the expression of CD133 in CRC cells contributed to increased stemness enhancement (Supplementary Figure S6). Furthermore, downregulation of CD133 markedly abrogated the inhibitory effect of SLNT on HCT116 cells, whereas overexpression of CD133 potentiated the inhibitory efficacy of SLNT. This phenomenon was further validated by the observation that, following SLNT treatment, cell viability was significantly reduced in the CD133-overexpressing SW480 group (OECD133 SW480) compared with that in the empty vector-transfected SW480 group (OENC SW480) (Figures 4C,D). Then, we sorted HCT116 and SW620 cells by flow cytometry and divided them into CD133^-^ and CD133^+^ fractions. Figure 4E clearly shows that, following treatment with SLNT at the same concentration, the viability of CD133^+^ CRC cells were significantly reduced compared to that of their CD133^-^ counterparts. Above results explained that CRC cells with high levels of CD133 are more susceptible to the cytotoxic effects of SLNT. Notably, CD133^+^ colorectal cancer cells are well-recognized to possess stemness properties. Therefore, the results presented in Figure 4E further support that SLNT exerts a potent stemness-inhibitory effect on CRC cells.

*In vivo* experiments, as shown in Figure 4F, both SLNT treatment and CD133 silencing resulted in a more pronounced suppression of tumor growth in nude mice relative to the shNC group. However, the tumor-suppressive effect of SLNT was notably impaired by CD133 silencing. Similarly, the results in SW480 tumor-bearing mice were consistent with the *in vitro* findings.

Here, we further validated the tumorigenicity of CD133 and showed that SLNT exhibits a stronger suppressive effect on CD133^+^ CRC cells with respect to their oncogenic features. Taken together, the above findings imply that CD133 may serve as a critical target through which SLNT exerts its anti-cancer effects against CRC.

### 3.5 SLNT interacts with CD133 and inhibits its expression

**FIGURE 5:**
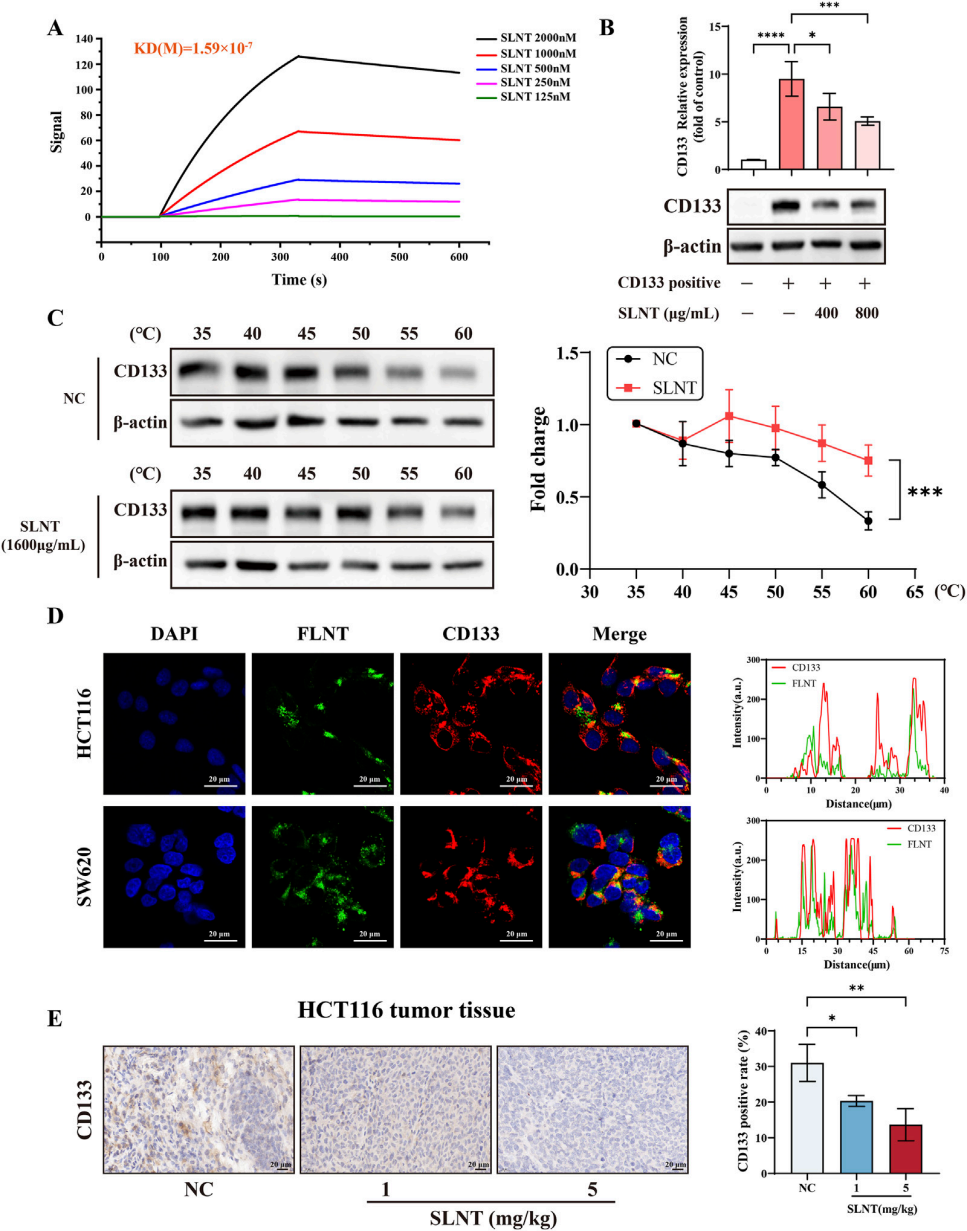
SLNT directly binds to CD133 and has a stronger tumor-suppressive effect on CD133^+^ CRC cells. LSPR **(A)** and colocalization images in HCT116 and SW620 cells **(D)** between CD133 and SLNT. HCT116 cells, treated with or without SLNT (1,600 μg/mL) for 6 h, were lysed and the resulting whole cell lysates were subjected to the CETSA to evaluate the thermal stability of CD133 **(C)**. SLNT treated CD133 expression in CD133^-^ and CD133^+^ HCT116 cells and HCT116 tumor tissues with or without SLNT treatment **(B,E)**. n ≥ 3, ^*^ *p* < 0.05, ^**^ *p* < 0.01, ^***^ *p* < 0.001, ^****^ *p* < 0.0001, ns, no significance.

Previous studies have indicated that small molecule drugs can directly bind to Gli1 within the stemness pathway and suppress its expression, thereby inhibiting stemness and exerting anti-cancer effects (Chen et al., 2022). However, whether polysaccharides exhibit a similar mechanism remains unclear. To investigate whether CD133 may serve as a potential target of SLNT in CRC, we incubated HCT116 cells with 5-DTAF-labeled lentinan (FLNT) at 4 °C and analyzed the interaction by flow cytometry. The results demonstrated that macromolecular SLNT could be internalized by HCT116 cells even at low temperature, suggesting a direct interaction between SLNT and the cells (Supplementary Figure S8). This uptake may be mediated by specific recognition of SLNT by surface receptors or active molecules on HCT116 cells (Hu et al., 2023). Then LSPR and CETSA were performed. As shown in Figure 5A SLNT specifically bind to CD133 conjugated on the chip with a dissociation equilibrium constant of 1.59 × 10^−7^ M, whereas β-glucan binds to Dectin-1 or TLR4 with a KD value of 5.08 × 10^−6^ or 4.6 × 10^−6^M (Yang et al., 2018, p. 4; Wu et al., 2021), indicating that SLNT has a stronger binding capacity for CD133 than Dectin-1 and TLR4. Moreover, colocalization of SLNT with CD133 was demonstrated by immunofluorescence in HCT116 and SW620 cells, as shown in Figure 5D. Furthermore, CETSA with HCT116 cells revealed that SLNT significantly enhanced the thermal stability of the CD133 protein (Figure 5C), indicating that SLNT could interact with CD133 in HCT116 cells. Next, we sorted HCT116 and SW620 cells by flow cytometry and divided them into CD133^-^ and CD133^+^ fractions. Figure 5E clearly showed that CD133^-^ CRC cells exhibited a markedly higher viability compared to CD133^+^ CRC cells after treatment with the same concentration of SLNT. It is worth mentioning that CD133^+^ CRC cells were considered to exhibit stemness characteristics. Therefore, the results in Figure 5E also reflected the strong stemness-inhibitory effect of SLNT on CRC cells. In short, results above indicated that CD133 is likely to be the anti-CRC target of SLNT.

To assess the impact of SLNT on CD133 expression, we measured CD133 levels *in vitro* and *in vivo* after SLNT administration. SLNT markedly decreased CD133 protein expression in HCT116 and CD133^+^ HCT116 cells (Figure 5B; Supplementary Figure S9). Similar results were shown in HCT116 tumor tissues using IHC and Western blot assays (Figure 5E; Supplementary Figure S9). Moreover, we found the mRNA level of CD133 in HCT116 cells was also decreased by SLNT treatment (Supplementary Figure S10).

### 3.6 Simulation of the binding mechanism of SLNT with CD133 via molecular docking

**FIGURE 6:**
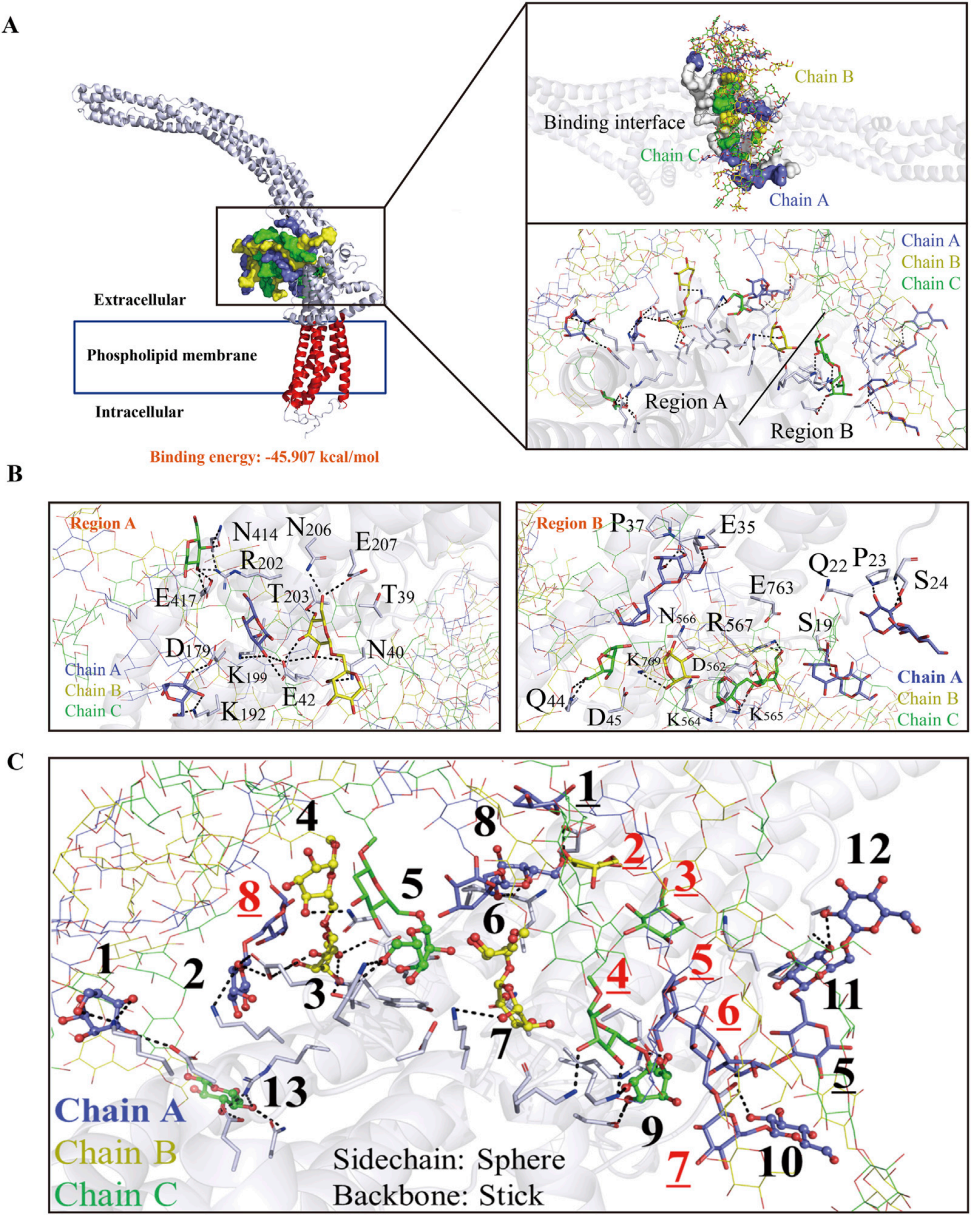
Molecular docking simulation between SLNT and CD133. Schematic diagram of the binding between SLNT and CD133 protein **(A)** and binding site **(B)**. Refinement analysis of the binding patterns between the main (black) and side chains (red) of SLNT and CD133 protein **(C)**.

Although we found that SLNT could bind specifically to CD133, the mechanism of their binding was unknown. Molecular dynamics simulations have become an important research method for elucidating biological mechanisms and have important applications in the discovery of drug targets. Therefore, we attempted to construct the structures of SLNT and CD133 via computers and conduct molecular docking simulations with the aim of identifying binding sites between them. The results (Figures 6A,B) showed that the lowest binding energy calculated for docking SLNT and CD133 was −45.907 kcal/mol, indicating a strong binding capacity, which confirmed the LSPR results. Further analysis of the interbinding patterns of SLNT and CD133 revealed that a variety of amino acids including asparagine (Asn) (40,414,206,566), glutamic acid (Glu) (207, 417, 42, 35, 763), arginine (Arg) (202, 567), threonine (Thr) (39, 203), lysine (Lys) (199, 192, 769, 564, 565), aspartic acid (Asp) (179, 45, 562), proline (Pro) (23, 37), serine (Ser) (19, 24), glutamine (Gln) (22, 44), in the extracellular region of the CD133 protein within a binding distance of 4.5Å could form a good hydrogen bonding network system with SLNT. In addition, according to Figure 6C, in the direct contact interface formed between triple helix SLNT and CD133 protein, the side chain region of SLNT mainly bound to amino acids in the extracellular domain of the CD133 protein. There were total of 13 binding amino acid sites in this region, and there were fewer binding amino acid sites between the main chain of SLNT and the CD133 protein, with a total of 8. This result was a consequence of the side chains of SLNT, which were located on the surface of the triple helix structure, exerting a steric hindrance effect, that prevented more than minimal exposure of the internal main chain. In contrast, the side chains could bind to a greater extent to the CD133 protein. Interestingly, when the 1,6-linked branched chain in SLNT became shorter, the lowest binding energy of the polysaccharide to CD133 increased from −45.907 kcal/mol to −37.981 kcal/mol, indicating a weakened binding ability (Supplementary Figure S11). As reported in the literature (Liu et al., 2018; Huang and Huang, 2021), the above results to some extent revealed that the spatial structure and the number of branched chains of SLNT might influence their antitumor activity.

### 3.7 SLNT negatively regulates the CD133/p85/p-AKT signaling axis, thereby inhibiting the stemness of CD133^+^ CRC cells

CD133^+^ cancer cells are considered to possess certain tumor stem cell characteristics, so they are often used in research on the efficacy and mechanism of drugs in treating CSCs (Gisina et al., 2023). In addition to its biological functions, CD133 serves as a key regulatory molecule, modulating signal transduction networks essential for tumorigenesis. CD133^+^ cells in CRC and gastric cancer exhibited a hyperactivated AKT signaling state, in contrast to the CD133^-^ cells (Zhao et al., 2015). CD133 has been reported to interact with p85 and promote tumorigenicity in glioma cells (Wei et al., 2013). In CRC, overexpression CD133 activates AKT and prevents cancer cell death (Mori et al., 2021). Our previous studies have demonstrated that SLNT can significantly inhibit the phosphoinositide 3-kinase (PI3K)/AKT signaling pathway and the expression of CD133 protein in HT-29 cells with a relatively high CD133^+^ cell proportion (Supplementary Figure S12) (Wang et al., 2016). The binding of SLNT and CD133 was demonstrated in this study, here, we suspected that SLNT might also influence the CD133/p85/p-AKT signaling axis in CRC.

**FIGURE 7:**
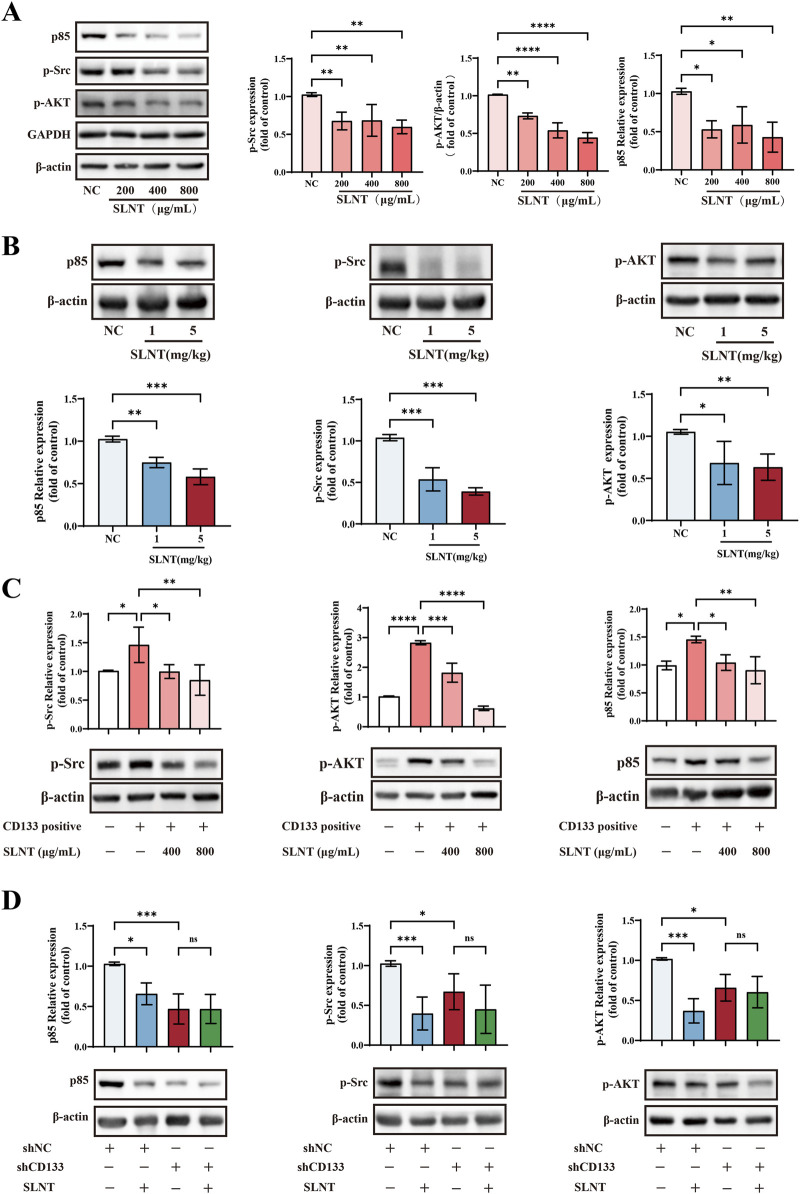
SLNT negatively regulates the CD133/p85/p-AKT signaling axis *in vivo* and *in vitro*. The protein expression levels of p85, p-Src, p-AKT were detected by Western blot assay in HCT116 cells with or without 200, 400, 800 μg/mL SLNT treated **(A)**, CD133^-^ HCT116 cells, CD133^+^ HCT116 cells and 400, 800 μg/mL SLNT treated CD133^+^ HCT116 cells **(C)**, and in tumor tissues of HCT116 and shNC/shCD133 HCT116 xenograft model **(B,D)**. n = 3, ^*^ *p* < 0.05, ^**^ *p* < 0.01, ^***^ *p* < 0.001, ^****^ *p* < 0.0001, ns, no significance.

**FIGURE 8:**
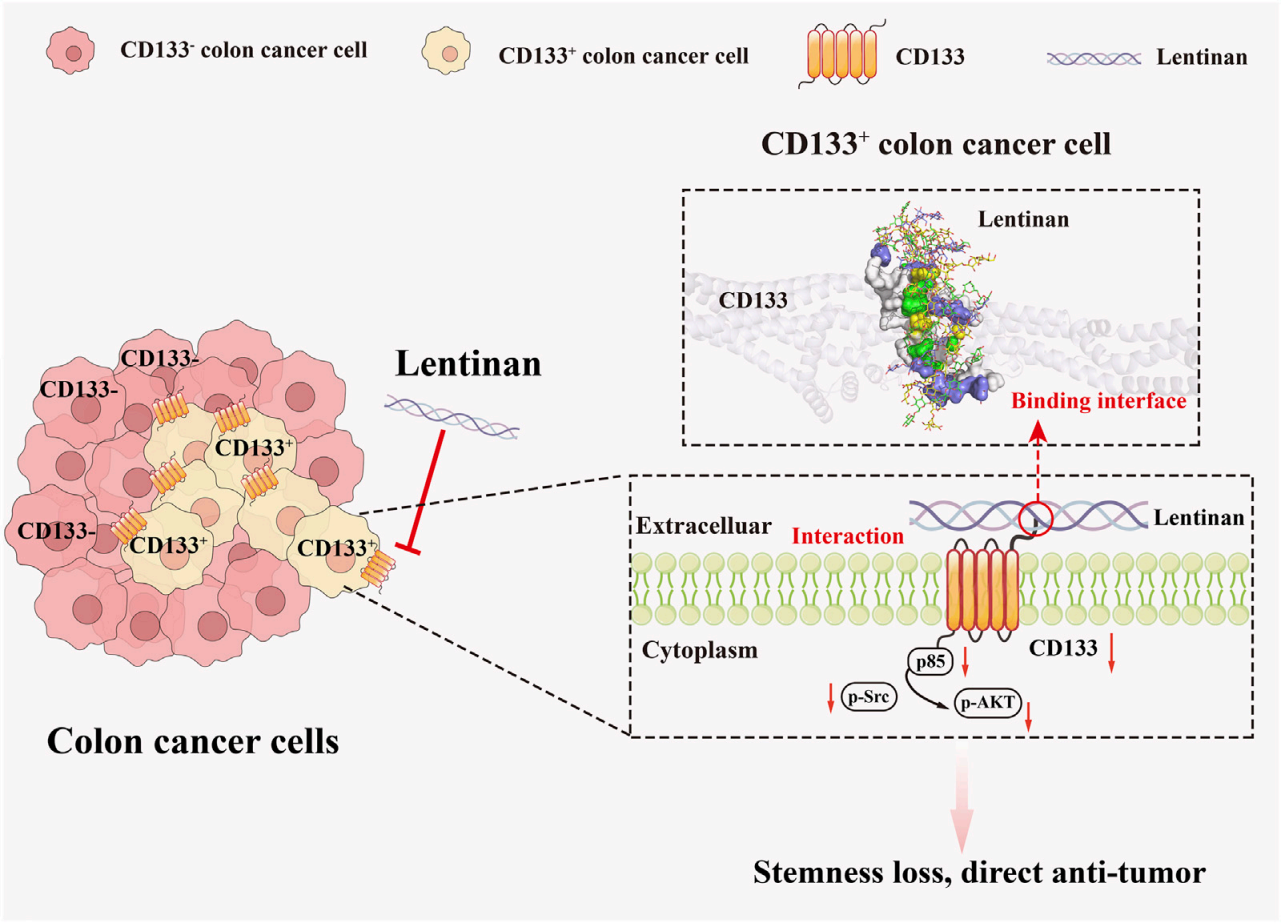
Mechanism diagram of SLNT inhibits human CRC growth.

According to Figures 7A,B, a dose-dependent decreased in p85, p-Src, and p-Akt levels was observed in both HCT116 cells and corresponding xenograft tumors following treatment with SLNT. We further separated the HCT116 CRC cell line into distinct CD133^+^ and CD133^-^ populations, and combined this with a CD133-knockdown HCT116 cells xenograft model to investigate the potential regulatory relationship between CD133 and p85/p-AKT following SLNT treatment. Results revealed that CD133^+^ cells exhibited high-expression of p85, p-Src and p-AKT compared to CD133^-^ cells, suggesting a positive correlation between CD133 expression and p85/p-AKT signal axis. However, treatment with 400 μg/mL and 800 μg/mL SLNT markedly downregulated p85, p-Src and p-AKT expression in CD133^+^ HCT116 cells (Figure 7C). Furthermore, in tumor tissues, knockdown of CD133 led to significant suppression the protein expression levels of p85, p-Src and p-AKT. Notably, the inhibitory effect of SLNT on these proteins were attenuated upon CD133 knockdown (Figure 7D). Collectively, we suspected that SLNT exerted anti-CRC stemness effects by suppressing CD133 expression, thereby negatively regulating the downstream p85/p-AKT signaling axis (Figure 8).

## 4 Discussion

Colorectal cancer stem cells (CCSCs), particularly the CD133^+^ subpopulation, are pivotal in tumor recurrence, metastasis, and resistance to therapy (Fang et al., 2010). However, targeted therapeutic strategies against these cells remain limited. In this study, we assessed the inhibitory effects of lentinan on the stemness properties of CD133^+^ CRC cells and explored the underlying molecular mechanisms. Lentinan has attracted extensive attention due to its remarkable anti-tumor activity (Ren et al., 2012; Meng et al., 2020). Over the years, the mechanism by which lentinan exerts its anti-tumor effects is increasingly understood. Current reports indicate that lentinan mainly exerts its anti-cancer effects through three major approaches. First, by eliciting potent antitumor immune effector responses (Hashimoto et al., 2002). Second, by directly inducing apoptosis or autophagy in tumor cells (Li et al., 2018; Hu et al., 2023). And third, by inhibiting tumor angiogenesis, thereby restricting tumor growth (Deng et al., 2018). Our initial investigations centered on the direct killing mechanism of SLNT on colorectal cancer (CRC) (Zhang et al., 2021), without involving aspects related to CRC stemness. Several reports have indicated that β-glucan have potential for CSCs treatment. For example, drug-carrying nanoparticles modified with 1,3-β-glucan exhibit enhanced efficacy in suppressing glioma stem cells compared to their unmodified (Singh et al., 2018). Additionally, β-glucan in combination with aspirin has been shown to reduce CD133 expression in lung cancer (Lu et al., 2019). However, studies above are largely confined to phenomenological observations of drug efficacy, with limited exploration of the underlying mechanisms. Crucially, the specific impact of β-glucan on CCSCs has not been previously established. In this work, we report for the first time that lentinan, a typical β-glucan suppresses stemness in CRC cells, and we investigated its mechanism through multiple approaches.

Accumulating evidences indicate that polysaccharides can bind to tumor-associated proteins to be functional recently. For instance, polysaccharides have been shown to target metabolic enzymes such as an α-D-glucan binding to ALDOA or other functional proteins such as safflower polysaccharide HH-1 targeting galectin-3 to inhibit tumor growth (Yao et al., 2019; Wang et al., 2021). While the binding potential of β-glucan to receptors like Elongation factor 1-alpha (eEF1a1) has been mentioned (Zou et al., 2019), its mechanistic depth, particularly concerning CSCs, remains largely unexplored. Our study first demonstrated that SLNT can bind to the CCSCs marker CD133 protein and inhibit its protein expression, thereby exerting an effect on suppressing stemness. Although the mechanism by which SLNT inhibited CD133 protein expression remains unclear, based on the finding that CD133 mRNA expression is also inhibited by SLNT, we suspected that the SLNT-CD133 interaction might activate intracellular signaling pathways that suppress transcription factors essential for CD133 gene expression, thereby reducing both mRNA synthesis and protein translation.

Polysaccharide-binding receptors have been studied to a greater extent in immune cells and less in tumor cells (Meng et al., 2020). Dectin-1, a C-type lectin like pattern recognition receptor (PRR), is considered an accepted β-glucan receptor. The carbohydrate recognition domain (CRD) is the key point at which Dectin-1 and β-glucan specifically bind (Brown, 2006; Dulal et al., 2018). Molecular dynamics simulations of lentinan and Dectin-1 were performed in our previous study. The side chains of lentinan were easily inserted into the groove of Dectin-1, indicating that they might contribute to specific binding (Wu et al., 2021). However, we did not find a similar pocket structure in the binding simulations of CD133 and SLNT. According to our current results and literature reports (Manabe and Yamaguchi, 2021), we can only conclude that the distinctive triple helix structure of SLNT facilitated its concentrated binding to the CD133 protein, while the abundant hydroxyl groups along the sugar chain endowed SLNT with a robust affinity for polar amino acids on the protein surface, thereby enhancing its interaction capabilities. This physical interaction suggests that the binding of SLNT to CD133 is not restricted by tumor type, but may occur in all cells expressing CD133. As for whether this binding is specific and whether SLNT can bind to other membrane proteins and affect other signaling pathways, further research is needed in our future studies.

CD133 is a CRC stem cell marker that also has a molecular function. Existing evidence indicates that deletion of CD133 inhibits p-AKT, and that AKT suppression effectively targets CSCs (Alobaidi et al., 2025). This is in line with our discovery that SLNT attenuates CRC stemness through the CD133/p85/p-AKT signaling axis. Although the exact mechanism requires further investigated, the consistency between our study and prior evidence suggests that inhibiting the CD133/AKT signaling axis is a viable therapeutic strategy against CSCs. Clinically, our findings may be conducive to reducing the risk of drug resistance and recurrence in CRC patients, and also provides a basis for broadening the clinical application of SLNT. Emerging evidence from the current study indicates that CD133 predominantly exerts its physiological functions via interactions with other molecules. It has been reported that CD133 could interact with p85 and promote tumorigenicity in glioma cells. CD133-p85 interaction was disrupted by LDN193189 and inhibited phosphorylation of AKT in liver tumor-initiating cells as well (Wei et al., 2013; Liang et al., 2021). Reports revealed that CD133 physically and functionally interacts with EGFR, facilitating AKT pathway activation in hepatocellular carcinoma and pancreatic cancer (Weng et al., 2016; Jang et al., 2017). Thus, we reasonably hypothesize that in CRC, SLNT most likely inhibits the downstream AKT signaling axis by impeding the interaction between CD133 protein and p85, thereby exerting its anti-cancer effect. In our future research, we will continue to investigate potential direct interacting molecules of CD133 using co-immunoprecipitation (Co-IP) or proximity ligation assay (PLA).

In current research on pharmacotherapy for CRC, the core objective lies in enhancing therapeutic efficacy and reducing adverse effects (Li et al., 2024c). In this context, emerging strategies such as immunotherapy and chemodynamic therapy have drawn considerable attention, among which novel delivery systems represented by nanotechnology exhibit broad prospects (Li et al., 2024a; Yan et al., 2024; Zu et al., 2024). However, the clinical translation rate of nanomedicines and their long-term safety remain major challenges to be addressed. Therefore, the development of low-toxicity natural drugs or the expansion of new indications for pharmaceuticals that have been clinically used emerges as a feasible pathway to improve the efficiency of clinical translation. For example, the natural product quercetin has been confirmed to sensitize the chemotherapeutic agent 5-fluorouracil (5-FU) for CRC treatment (Li et al., 2024b), and Glasdegib (PF-04449913), which is clinically used to treat acute myeloid leukemia (AML), has also shown therapeutic efficacy against CRC (Ruan et al., 2024). This study focuses on lentinan, a drug with years of clinical application, high safety profile, and well-defined antitumor activity (Zhou et al., 2024). The specific molecular mechanism of its anti-CRC effect is worthy of in-depth exploration, which is of great significance for maximizing its clinical application value.

Our study not only provides new possibilities for understanding the anticancer mechanisms of SLNT, but also demonstrated SLNT has potential to expand clinical applications in two key directions. First, the ability of SLNT to inhibit tumor stemness, when combined with standard chemotherapy, can synergistically inhibit the recurrence and metastasis of CRC. Second, the ability of SLNT to bind CD133 provides a new treatment strategy for microsatellite stable (MSS) CRC that is resistant to immunotherapy. Furthermore, the advancements in microbiomics have revealed the significant potential of the gut and intratumoral microbiota in the diagnosis and treatment of CRC (Gou et al., 2024; Zhang et al., 2024). Notably, studies have confirmed that lentinan has a remarkable regulatory effect on gut microbiota dysbiosis (Ji et al., 2022; Zhang et al., 2025). Therefore, exploring the anti-CRC mechanism of SLNT from the microbiome perspective represents a highly promising direction for subsequent research extensions.

While our findings support the hypothesis that the CD133/p85/p-AKT axis in SLNT-mediated suppression of cancer stemness, several limitations should be considered. Firstly, the precise structural basis of the interaction between SLNT and the CD133 protein remains to be fully elucidated. Although this study predicted the potential binding sites between SLNT and CD133 protein via molecular docking, providing a structural reference for their interaction, this method has limitations. For instance, docking simulations, based on static structural analysis, cannot fully reflect the impact of dynamic conformational changes of intracellular proteins and SLNT on the actual binding mode. Therefore, subsequent studies should incorporate long-term molecular dynamics simulations or dynamic interaction analyses to more accurately reveal their binding mechanism. Secondly, in animal models, we found no significant difference in the inhibitory effect on tumors between 1 mg/kg and 5 mg/kg SLNT, and there was no dose-dependent effect *in vivo*. As polysaccharides with a molecular weight greater than 40 kDa may exhibit nonlinear elimination kinetics *in vivo* (Mehvar et al., 1995; Kaneo et al., 1997), we speculate that SLNT may have reached its optimal therapeutic window within the dose range of 1-5 mg/kg. This might be the reason why no significant difference in efficacy was observed between the 1 mg/kg and 5 mg/kg dose groups. Therefore, we will focus on evaluating the pharmacokinetic characteristics of SLNT *in vivo* in the future. Finally, our *in vivo* findings are based on xenograft models, which do not fully recapitulate the human tumor microenvironment context. We will conduct experiments using patient-derived xenograft (PDX) models to further verify the inhibitory effect of SLNT on tumor stemness and its underlying mechanisms.

## 5 Conclusion

In summary, this study reveals that SLNT exhibits potential inhibitory effects on CCSCs. By binding and inhibiting CD133, SLNT attenuates the stemness properties of CD133^+^ CRC cells and suppresses the CD133/p85/p-AKT signaling axis. These findings establish a basis for the development of SLNT as a promising anticancer agent for future therapeutic and pharmaceutical applications.

## Data Availability

The raw data supporting the conclusions of this article will be made available by the authors, without undue reservation.
