# Supplementary material for: Lentinan inhibits colorectal cancer stemness by binding CD133 and suppressing the CD133/p85/p-AKT signaling axis
Source: Front Pharmacol. 2025 Dec 10;16:1725716. doi: 10.3389/fphar.2025.1725716 (PMC12727974; doi:10.3389/fphar.2025.1725716)
Supplement: Supplementary file 1 [file Supplementaryfile1.docx]

# Supplemental Information

# Lentinan inhibits colorectal cancer stemness by binding CD133 and suppressing the CD133/p85/p-AKT signaling axis

# Supplemental methods

- 1. **Preparation of Lentinan**

Lentinan was extracted, isolated and purified from the dried fruit bodies of Lentinus edodes originating from Fangxian (Hubei Province, China) through a series of methods detailed by our previous study (Wang et al., 2013). In short, the dried fruit bodies of L. edodes were soaked in water, softened, washed, and cut into small pieces. Next, after soaking the pieces in 95 % alcohol overnight to remove organic impurities, Lentinus edodes was boiled twice in 2L hot water for 1h each time. Then, heated and concentrated the extraction solution to 600ml, and added an equal volume of 95% ethanol overnight after cooling the concentrated solution. Then, the alcohol precipitation solution was centrifuged at 4000 rpm for 15 min and the sediment was collected and dried. Subsequently, the crude polysaccharide was decolored with 3% H_2_O_2_ at 55 ℃ for 5h and the precipitate was collected by centrifugation and dried after added an equal volume of 95% ethanol overnight. Finally, the decolored polysaccharide was dissolved in dilute alkaline solution and then ultrafiltration with a 300,000 ultrafiltration membrane cut-off ultrafiltration, the solution was collected, dialyzed and lyophilized to obtain the purify polysaccharide.

- 1. **Structure of SLNT**

The molecular weight, monosaccharide composition, triple helix structure, glycosidic bonds and their linkages were determined by HPGPC, UV spectrophotometry, HPLC, congo red assay and methylation analysis, respectively, as our study previously described (Pan et al., 2022; Zhang et al., 2023).

**1.3 Western blot assay**

Total proteins were extracted from tumor tissues and cells with tissue or cell lysis containing 1% PMSF, protease inhibitors, phosphatase inhibitors. Protein concentration was measured by BCA protein assay kit. Each protein sample was mixed with loading buffer and boiled for 10 min. Equal amount of protein samples were separated by 10% or 12% SDS-PAGE and then transferred to nitrocellulose membranes (Amersham Pharmacia Biotech, Buckinghamshire, UK). After blocking by 5% non-fat milk or 5% BSA in Tris-buffered saline Tween (TBST) for 1.5-2h, the membranes were incubated by primary antibodies (CD133, p-AKT, p85, p-Src, β-actin) overnight at 4 ℃. Next, the membranes were washed by TBST for three times and incubated with secondary antibodies for 1 hour at room temperature. Finally, the membranes were detected by ECL kit (Meilunbio, China) and visualization with an Automated Imaging System (Gene Gnome XRD, UK) after washing by TBST for 3 times. The quantification of grayscale values of the bands was evaluated by ImageJ software (National Institutes of Health, Bethesda, MD, USA).

**1.4 Immunohistochemical analysis**

Detailed operation procedure could be found in our previous study(Pan et al., 2022). Briefly, after the tumor tissues were embedded in paraffin sections, the sections were dewaxed and rehydrated for antigen repair. Endogenous peroxidase was blocked by H_2_O_2_. The Ki67 and CD133 antibodies were incubated at 4 ℃ overnight followed by the incubation of secondary antibodies. The positive areas stained with DBA and the nucleus stained with hematoxylin. Finally, results were interpreted under a white light microscope (Olympus Corporation, Japan). Image-Pro Plus 6.0 software (National Institutes of Health, Bethesda) was used for semi-quantitative analysis of positive areas.

**1.5 Immunofluorescence assay**

In brief, cells of climbing plate were fixed in 4% paraformaldehyde for 20min, washed with PBS and blocked with 2% BSA dissolved in PBS for 1 h. Cells incubated with CD133 antibody (Novus) at 37℃ for 1h. Then, cells were incubated with anti-mouse CoraLite594-conjugated secondary antibody (proteintech) for 1h at room temperature. Finally, DAPI was used for nuclear staining. Images were observed by a confocal laser scanning microscope (Leica, Germany).

1. **Supplemental results**

**
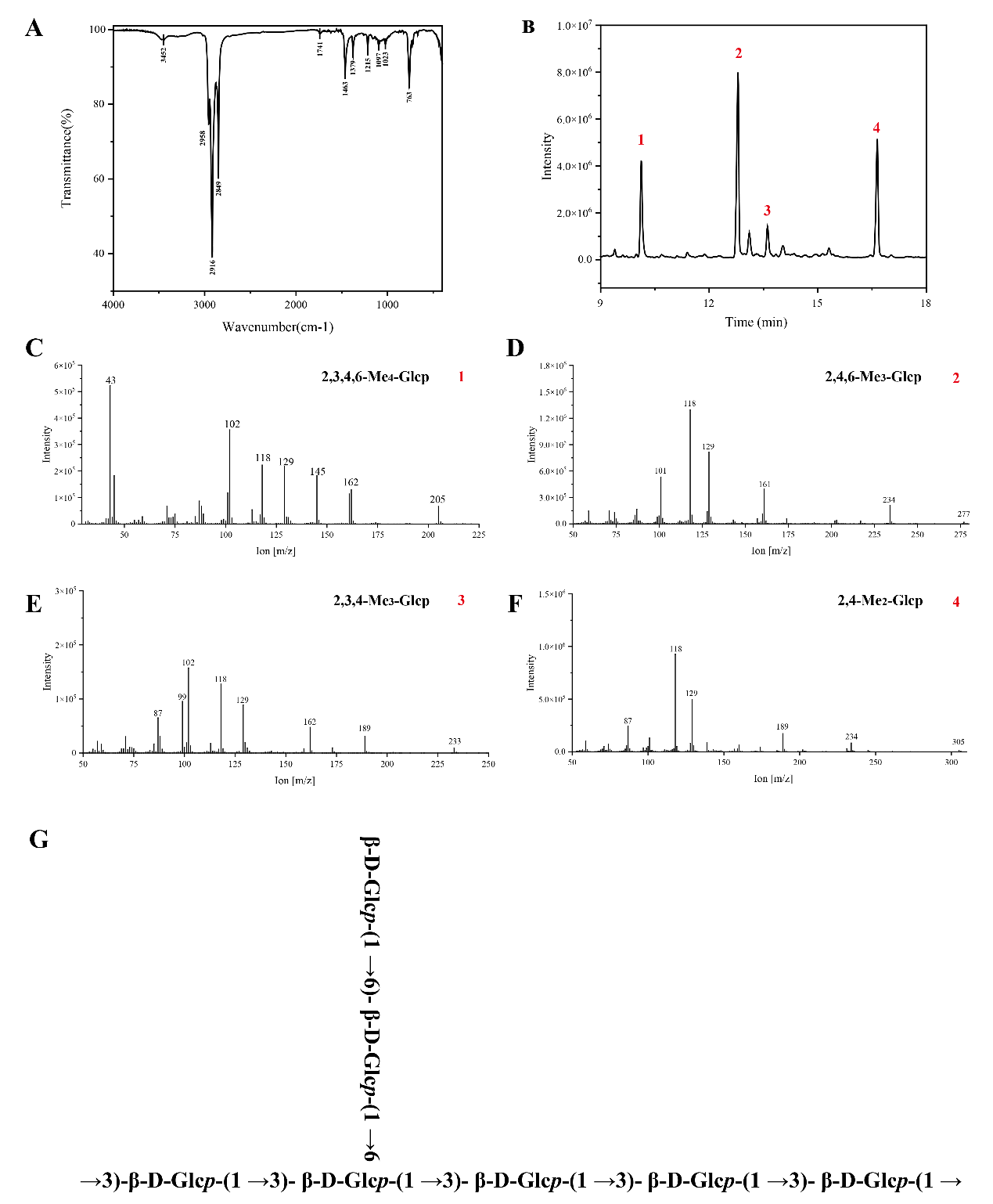
**

**Fig. S1: Results of methylation analysis of SLNT. Infrared spectrum of methylation products (A). Total ion chromatogram for methylation products of SLNT (A) and mass spectra for corresponding peaks (B-F). A proposed, putative repeating unit structure of SLNT (G).**

**Supplementary Table 1: Results of methylation analysis of SLNT**





**
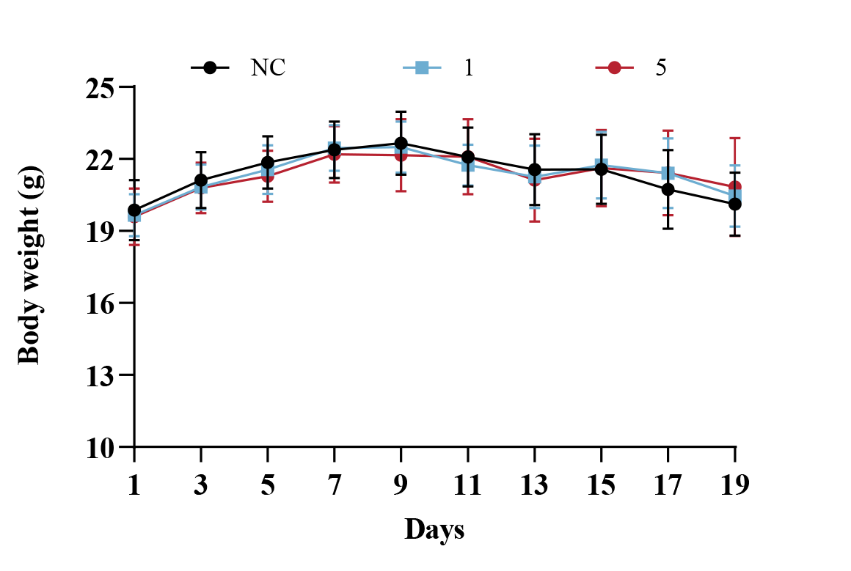
**

**Fig. S2 The body weight change curve of HCT116 tumor bearing mice during SLNT administration**

**
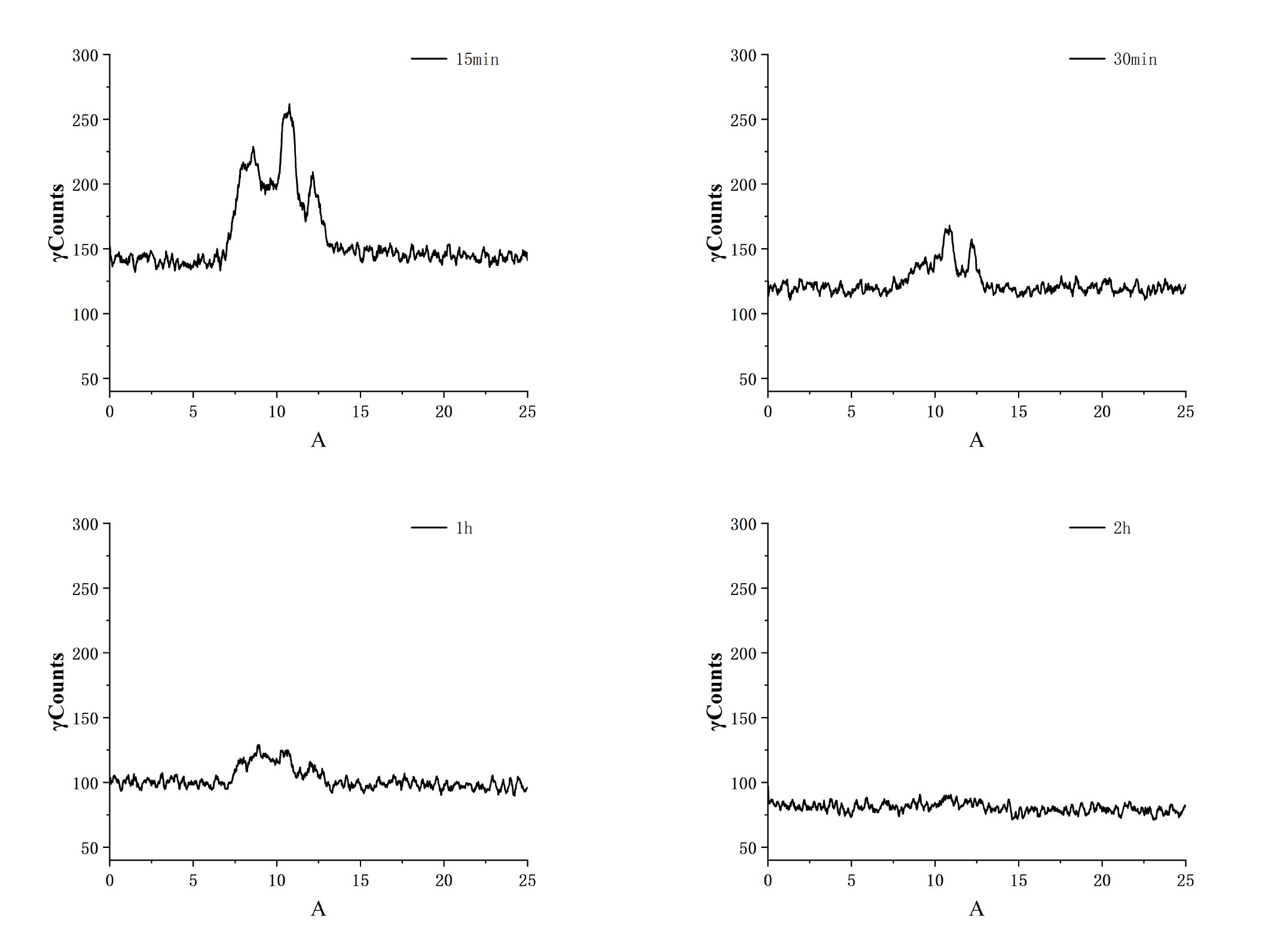
**

**Fig. S3: RadioHPGPC of tumor samples after intravenous administration of ^99m^Tc-LNT in HCT116 tumor-bearing nude mice.**

**
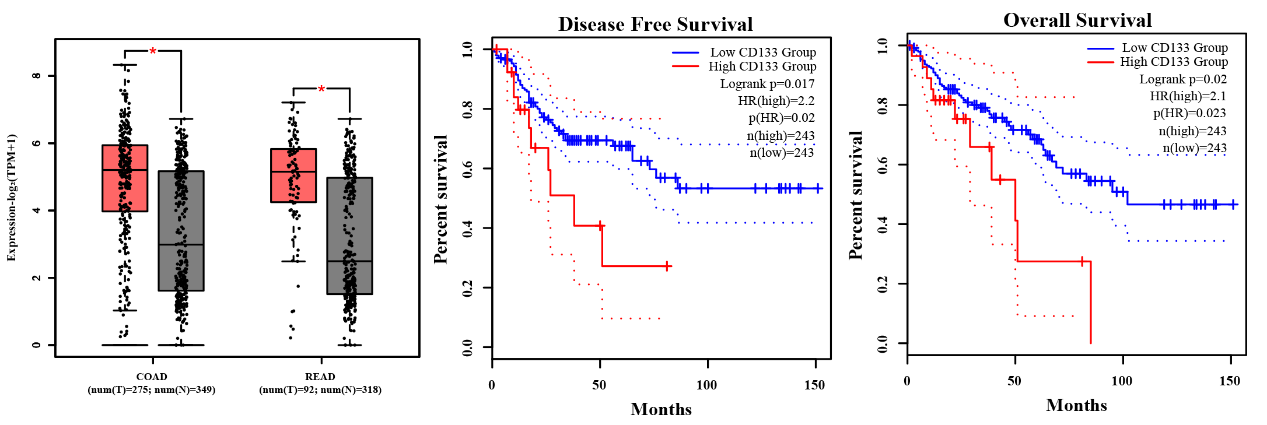
**

**Fig. S4: CD133 expression level in colorectal cancer and adjacent tissues, disease-free survival, and overall survival of colorectal cancer patients in GEPIA database.**

**
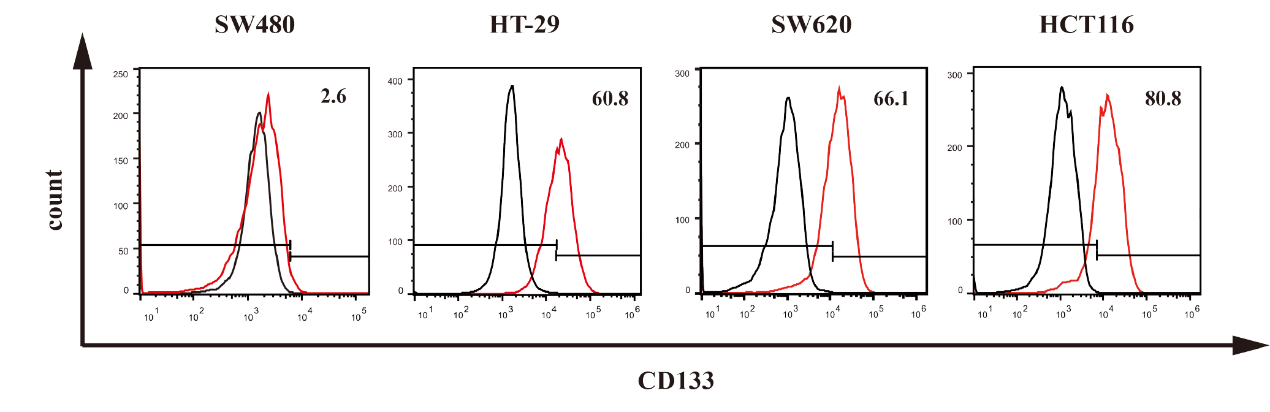
 Fig.S5: CD133^+^ cells proportion of SW480, HT-29, SW620, and HCT116 were detected by flow cytometric assay.**


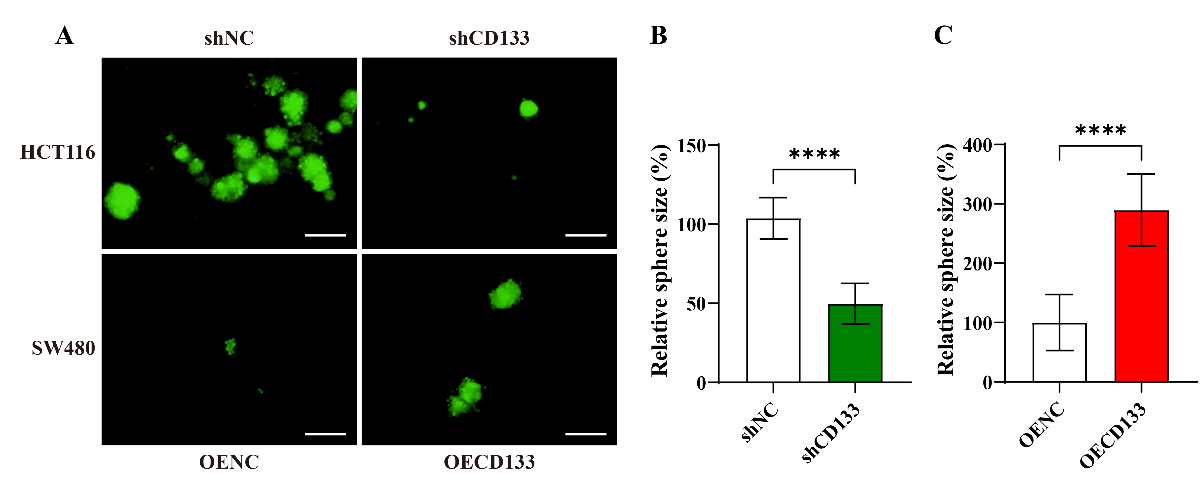


**Fig. S6: The effect of CD133 on the stemness characteristics of colon cancer cells.** Representative images of shNC/shCD133 HCT116 and OENC/OECD133 SW480 cell spheres (A) and diameter of stem cell spheres in each group (B, C). Scale bar: 500 μm.


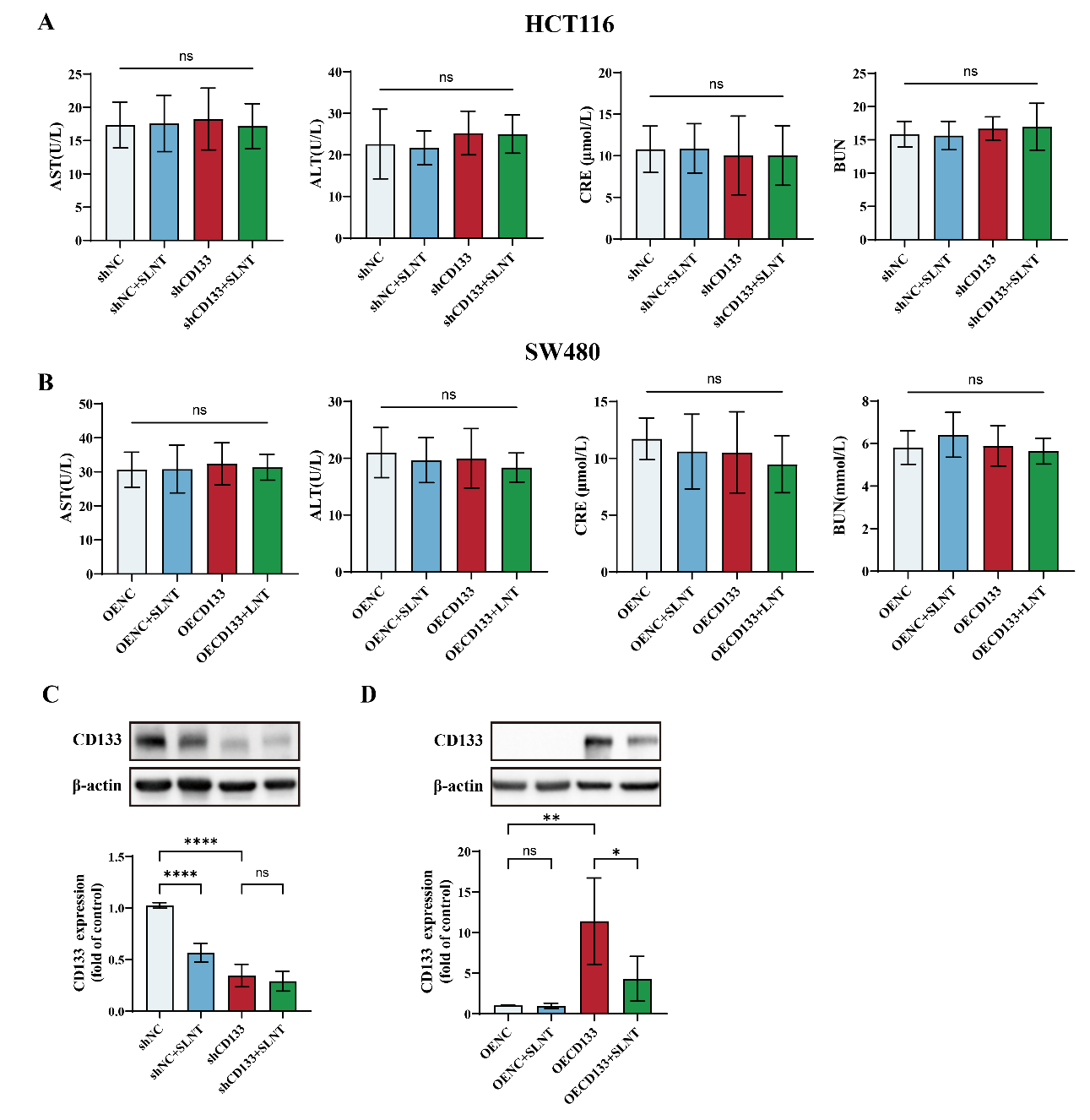


**Fig. S7:** **Serum AST, ALT, CRE, BUN expression levels in each group of shCD133 HCT116 (A) and OECD133 SW480 (B) tumor-bearing mice. CD133 expression levels in shCD133 HCT116 (C) and OECD133 SW480 (D) tumor tissues.**

**
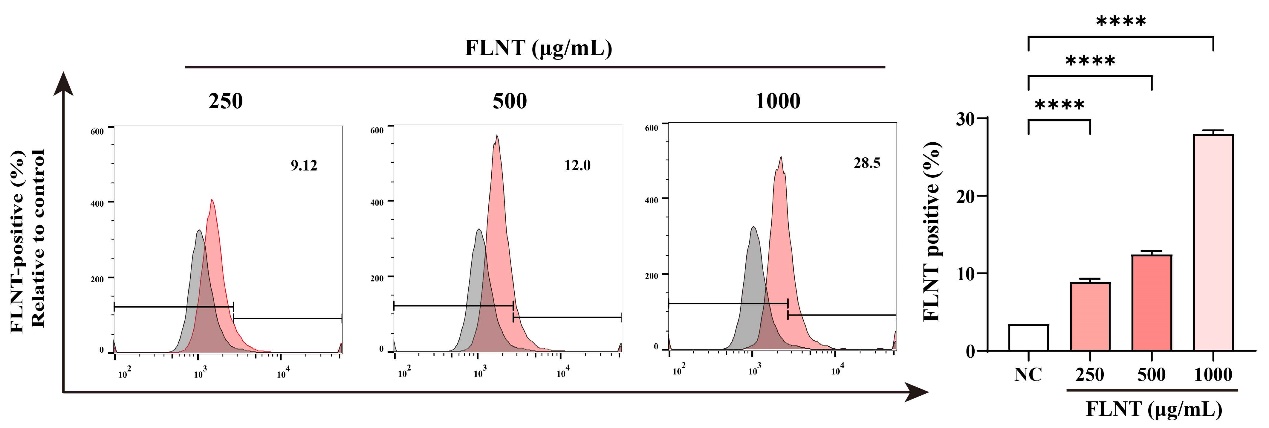
 Fig. S8:** **The flow cytometry analysis of HCT116 cells incubated with FLNT at different concentrations (250 to 1000 μg/mL) at 4°C.**

**
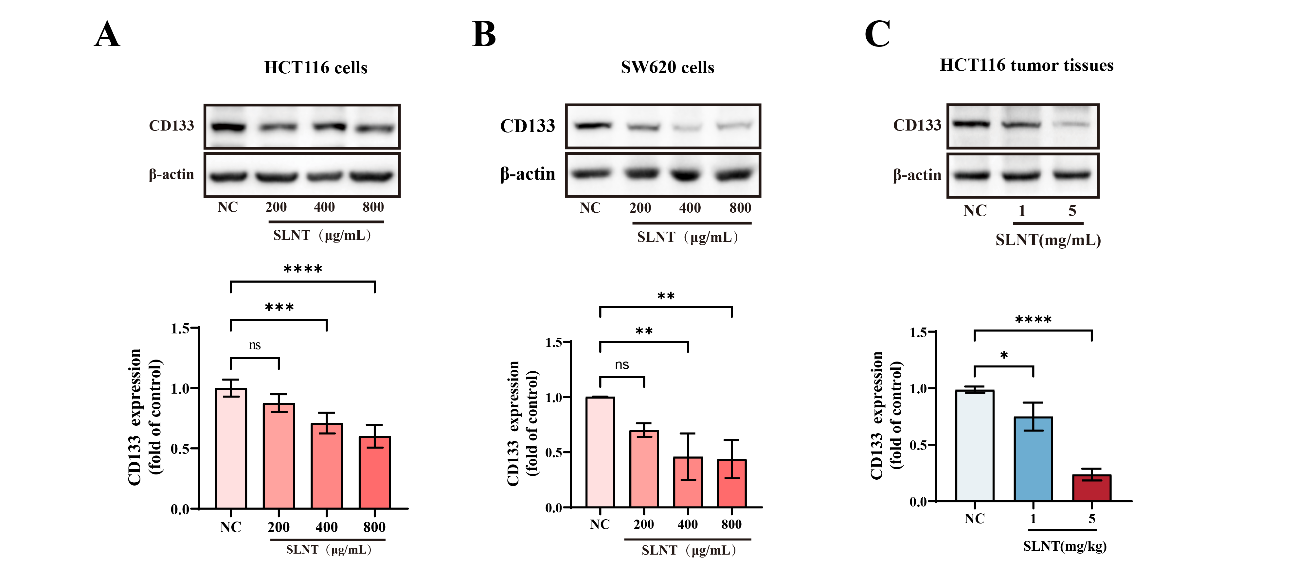
**

**Fig. S9: SLNT inhibited CD133 expression *in vitro* and *in vivo*.**

**
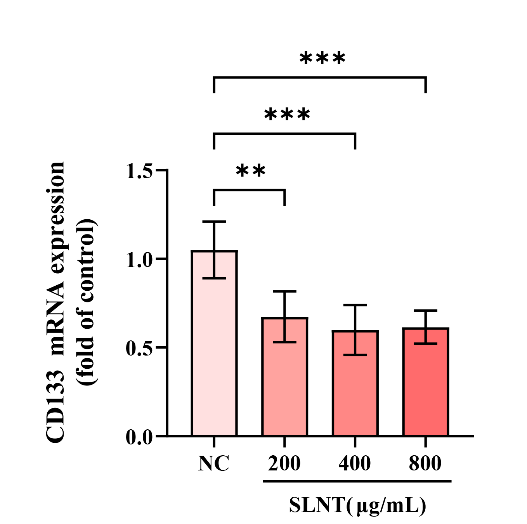
**

**Fig. S10: SLNT inhibited CD133 mRNA expression in HCT116 cells.**

**
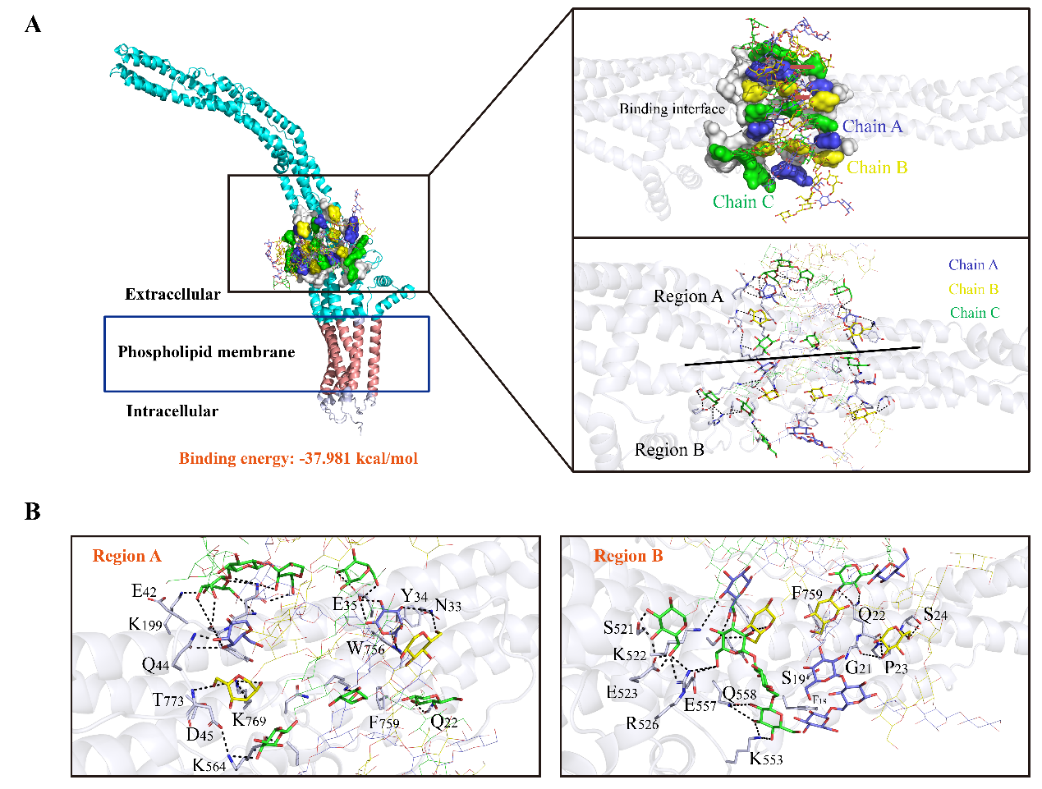
**

**Fig. S11: Molecular docking simulation between SLNT and CD133.** Schematic diagram of the binding between SLNT and CD133 protein (A) and binding site (B).

**
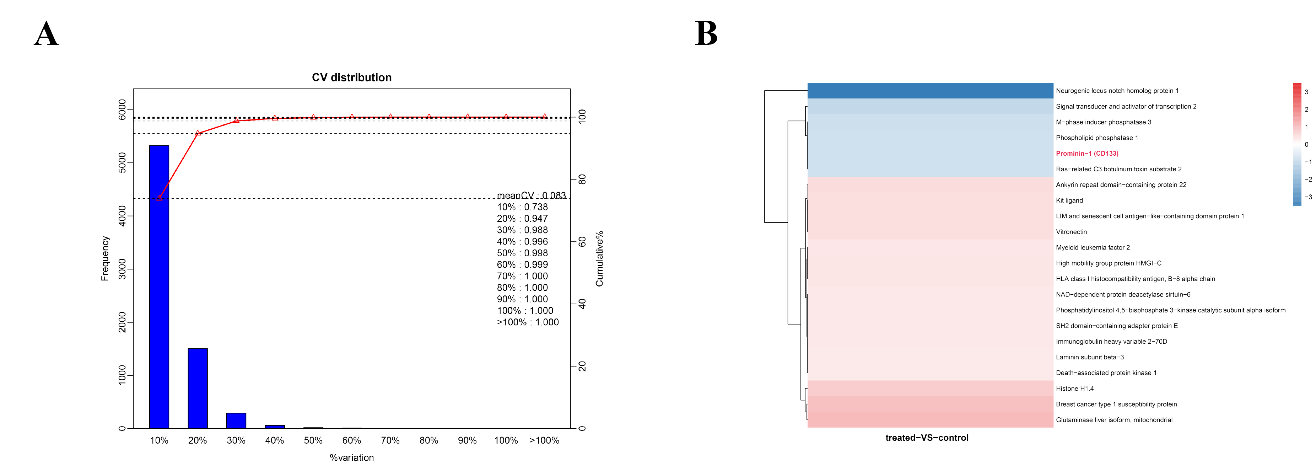
**

**Fig. S12: Proteomic detection of HT-29 cells treated with or without SLNT. Repeatability evaluation of iTRAQ protein quantitative analysis (A) Clustering heat map of tumor related proteins (B).**

**Reference**

Pan, X., Wang, H., Zheng, Z., Huang, X., Yang, L., Liu, J., et al. (2022). Pectic polysaccharide from Smilax china L. ameliorated ulcerative colitis by inhibiting the galectin-3/NLRP3 inflammasome pathway. *Carbohydr. Polym.* 277, 118864. doi: 10.1016/j.carbpol.2021.118864

Wang, K., Zhang, Q., Liu, Y., Wang, J., Cheng, Y., and Zhang, Y. (2013). Structure and Inducing Tumor Cell Apoptosis Activity of Polysaccharides Isolated from Lentinus edodes. *J. Agric. Food Chem.* 61, 9849–9858. doi: 10.1021/jf403291w

Zhang, Y., Tang, W., Zheng, Z., Nie, G., Zhan, Y., Mu, X., et al. (2023). Metabolic degradation of polysaccharides from Lentinus edodes by Kupffer cells via the Dectin-1/Syk signaling pathway. *Carbohydr. Polym.* 317, 121108. doi: 10.1016/j.carbpol.2023.121108
